# Meal Patterns and Food Choices of Female Rats Fed a Cafeteria-Style Diet Are Altered by Gastric Bypass Surgery

**DOI:** 10.3390/nu13113856

**Published:** 2021-10-28

**Authors:** Ginger D. Blonde, Ruth K. Price, Carel W. le Roux, Alan C. Spector

**Affiliations:** 1 Department of Psychology and Program in Neuroscience, Florida State University, Tallahassee, FL 32306, USA; blonde@psy.fsu.edu; 2 Nutrition Innovation Centre for Food and Health (NICHE), School of Biomedical Sciences, Ulster University, Coleraine BT52 1SA, UK; rk.price@ulster.ac.uk; 3 Diabetes Complications Research Centre, Conway Institute, School of Medicine, University College Dublin, D04 V1W8 Dublin, Ireland; carel.leroux@ucd.ie

**Keywords:** Roux-en-Y gastric bypass, cafeteria diet, meal pattern analysis, macronutrient selection, food choice, rat

## Abstract

After Roux-en-Y gastric bypass surgery (RYGB), rats tend to reduce consumption of high-sugar and/or high-fat foods over time. Here, we sought to investigate the behavioral mechanisms underlying these intake outcomes. Adult female rats were provided a cafeteria diet comprised of five palatable foodstuffs varying in sugar and fat content and intake was monitored continuously. Rats were then assigned to either RYGB, or one of two control (CTL) groups: sham surgery or a nonsurgical control group receiving the same prophylactic iron treatments as RYGB rats. Post-sur-gically, all rats consumed a large first meal of the cafeteria diet. After the first meal, RYGB rats reduced intake primarily by decreasing the meal sizes relative to CTL rats, ate meals more slowly, and displayed altered nycthemeral timing of intake yielding more daytime meals and fewer nighttime meals. Collectively, these meal patterns indicate that despite being motivated to consume a cafeteria diet after RYGB, rats rapidly learn to modify eating behaviors to consume foods more slowly across the entire day. RYGB rats also altered food preferences, but more slowly than the changes in meal patterns, and ate proportionally more energy from complex carbohydrates and protein and proportionally less fat. Overall, the pattern of results suggests that after RYGB rats quickly learn to adjust their size, eating rate, and distribution of meals without altering meal number and to shift their macronutrient intake away from fat; these changes appear to be more related to postingestive events than to a fundamental decline in the palatability of food choices.

## 1. Introduction

Roux-en-Y gastric bypass is a bariatric surgery designed to induce weight loss in patients with severe obesity. The procedure results in a substantial loss of body mass, which is maintained for many years along with the mitigation of typical obesity-associated comorbidities such as cancer, cardiovascular disease, and Type II diabetes mellitus [1,2,3,4,5,6,7,8,9,10,11]. The success of this surgery is often credited to the overall reduction in food intake. Patients report feeling less hunger and consuming fewer calories, especially foods high in sugar and fat [4,12,13,14,15,16,17]. There are also some indications that patients find the taste of these foods less appealing [18,19,20,21,22]. However, while self-reporting methods of measuring food intake have utility, they are vulnerable to mis-reporting, particularly under-reporting and are unlikely to be representative of usual food intake [23,24,25]. For example, a recent study found that when food choice and intake were measured directly in a buffet meal, patients with RYGB decreased their energy intake, but did not change their relative consumption of energy-dense foods. [3,26,27,28,29].

The altered gastrointestinal anatomy clearly leads to a variety of physiological changes that likely support a hypophagic pattern of behavior after RYGB. With the significant reduction in stomach capacity and the removal of control of gastric emptying via the pyloric sphincter, foods and fluids are deposited rapidly into the jejunum. A variety of gut hormone responses are altered after the procedure interacting with their cognate receptors in the periphery and potentially in the brain as well. It is typically reported that there is an increase in postprandial glucagon-like peptide-1 (GLP-1), peptide tyrosine tyrosine (PYY), and sometimes cholecystokinin (CCK); these are all hormones that have been shown to contribute to the termination of a meal (i.e., satiation; [30,31,32]). Some studies also indicate a reduction in ghrelin, which is a hormone associated with the initiation of a meal, but this has not been universally observed [33,34,35,36]. Many of these changes occur early after the surgery and well before there is substantial weight loss. Together, this new enteroendocrine profile would decrease overall intake by reducing the size of each meal and delaying the start of the next. Other physiological effects of the surgery that could potentially impact feeding behaviors include hypertrophy of the intestinal wall, malabsorption, altered microbiota profiles, and changes in bile acid secretion [37,38,39,40,41,42,43,44,45,46,47,48,49].

The systematic study of what food is consumed after RYGB and how it is eaten can help identify behavioral and physiological mechanisms that contribute to the success of the surgery. Rodent models are particularly useful in this regard because of the exceptional experimental control of theoretically relevant variables such as feeding conditions and provide a window into the fundamental physiological and behavioral processes affected by the surgery uncomplicated by cognitive, social, and economic factors associated with human food consumption (e.g., compliance with nutritional counseling; [50]), as relevant as those factors might ultimately be. Many of the basic consequences of RYGB on physiology and behavior in rats emulate those seen in humans; after the surgery both rats and humans lose body weight, have improved glycemic control, reduce intake, and display elevated levels of key gut hormones [18,51,52,53,54,55,56,57,58,59,60,61]. The changes in the relative intake of energy dense foods and fluids observed in rats after RYGB do not appear to be driven by an unconditioned modulation of the palatability of those foods [18,55,59,60,62,63,64,65,66]. In fact, the bases of the underlying changes in food selection after the surgery in rats, including whether those changes are tied to the postoral consequences of ingestion, remain unclear.

The quantification of the size and duration of meals and the timing between them, called meal pattern analysis, has been used for decades to provide insights into the underlying mechanisms and neural circuitry that influence nutrient intake [30,31,67,68,69,70,71,72,73,74,75,76], including after bariatric surgery [77,78,79,80]. However, it has also been shown that the food choices available ultimately influence feeding patterns [73,81,82,83,84]. For this reason, the use of a cafeteria-style diet, where multiple food choices are provided simultaneously and vary in their macronutrient content as well as other physical properties, has become more common in studies that use rodent models of human feeding behavior [85]. However, rodent meal pattern analysis has yet to be applied to a cafeteria diet paradigm or to understand if and how RYGB affects the intake and selection of complex food options in a rodent model. This is an important translational step in the analysis of the effects of the surgery because humans have a variety of food options at their disposal.

Accordingly, in this experiment, female rats were presented with an array of five foods while housed in a specialized apparatus that allowed the continuous measurement of the size and timing of meals. The direct quantification of food intake behavior demonstrated that, after RYGB surgery, rodents initially ate a meal similar in size as that of SHAM rats, suggesting that motivation to consume the diet was still high after surgery. However, by the second meal and continuing through the rest of post-surgical monitoring, RYGB animals consumed initiated smaller meals that were more evenly spread across the day. Food preferences changed more slowly across multiple days to reduce intake of fat but rats continued to consume most of their energy from foods high in fat and/or sugar. Taken together, these results suggest that when provided the opportunity to self-select foods, rats continue to be motivated to consume fat and sugar but instead adapt intake behaviors to more evenly spread energy consumption across the day, possibly in an attempt to avoid negative post-ingestive consequences of eating too much at one time.

## 2. Materials and Methods

### 2.1. Subjects

Because most bariatric patients are female [86,87], this study was conducted with thirty-two female Sprague-Dawley rats, aged 10–12 weeks upon arrival. Rats were housed in a facility where light (12 h light:12 h dark), temperature, and humidity were controlled automatically. All handling occurred during the light phase. Standard woodchip bedding was used throughout the experiment, except during recovery from RYGB surgery (see below). Upon arrival, rats were single-housed in standard polycarbonate cages and given ad libitum access to standard rat chow (LabDiet Rodent 5001; Purina, St. Louis, MO, USA), reverse-osmosis deionized water, and a stainless-steel toy for environmental enrichment (Rattle-A-Round, Otto Environmental; Greenfield, WI, USA). All procedures described here were approved by the Florida State University Animal Care and Use Committee (protocol 1817).

### 2.2. Apparatus

**Figure 1:**
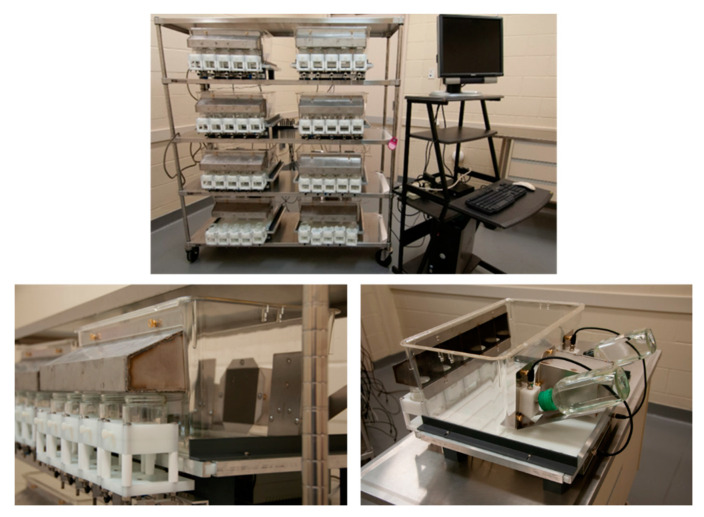
The five-Item Food Choice Monitor. Top: a set of customized cages connected to a computer. Bottom left: the front of a single cage, showing the components to measure food intake—the stainless-steel food hood, glass jars, HDPE jar holders and load cells to measure food intake. Bottom right: the back of a single cage, with the stainless-steel nest flanked by bottles set into the lick blocks, connected by cable to the interface box under the base of the unit.

All meal pattern monitoring was conducted in the five-Item Food Choice Monitor (FCM; Figure 1). Each individual cage unit is designed around a modified standard rectangular polycarbonate rodent cage (Tecniplast, Inc.; Montreal, Quebec, Canada) with access ports on either long side to allow custom stainless-steel pieces to be fit. On one long side of the cage, two Teflon lick blocks flank a stainless-steel nesting area (Figure 1, bottom right). These blocks hold the spouts of water bottles, and contain a small wire. The wire runs to a top-mounted BNC port so that a cable can connect the lick block to the interface circuitry. Licks to either bottle are registered when the tongue touches the spout and completes an electrical circuit.

The stainless-steel food hood is positioned opposite the water bottles (Figure 1, bottom left). The food hood extends out from the cage and has small holes cut into the base to allow access to five jars, and stainless-steel dividers to reduce cross-contamination. To reduce possible spillage, the holes are slightly smaller in diameter than the 4-oz glass food jars. The cage unit sits on an elevated base to allow placement of the jars under the hood. Aluminum angle stock is mounted across the front two legs of the base, and individual load beams that register jar weights protrude out from the front. A small custom aluminum bracket is attached to each load beam. The bracket holds the jar holder unit, comprised of multiple layers of High-Density Polyethylene. The bottom-most layer is oversized and has a routed interior to contain potential spillage on the load beam, maintaining the weight. The uppermost layers of the jar holder are smaller, and have circular space routed out to hold the jars. When in place, this entire unit is sized such that the top of the jar can be placed just under the bottom of the food hood.

Each cage unit has load beams and lick circuitry connected to an interface box that contains the controller board. The controller board is connected to a computer via USB, and data collection software records the weight (in mg) of each jar every 100 ms and each interlick interval (in ms).

### 2.3. Presurgical Measurements

#### 2.3.1. Presurgical Estrous Baseline

**Figure 2:**
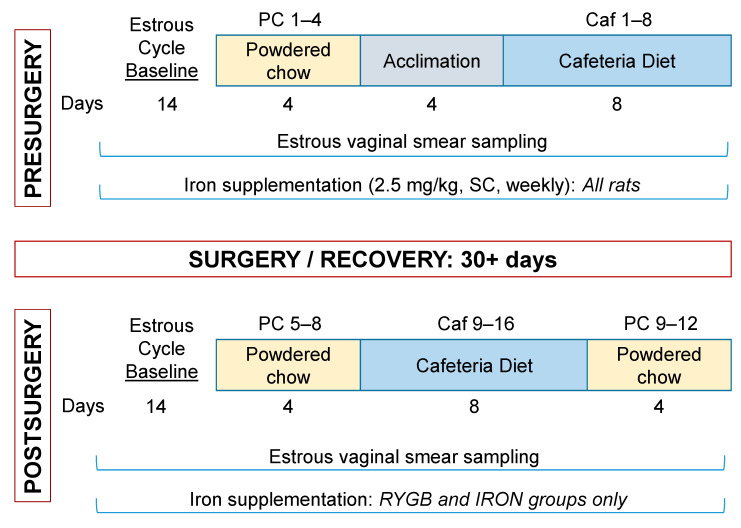
Experimental schedule.

A summary of the experimental schedule can be found in Figure 2. Rats acclimated to the facility for five days. Beginning on day 6 after arrival, daily vaginal swab samples were taken to determine phase of the estrous cycle. These continued throughout the experiment for 2 weeks preceding and throughout all food intake phases. Vaginal cytology was assessed daily by analyzing unstained samples under a light microscope. Also during this estrous baseline period, all rats began receiving prophylactic injections of iron dextran (2.5 mg/mL/kg, SC once weekly) to minimize the potential for iron deficiency after RYGB surgery. Rats in the RYGB or the iron-treatment nonsurgical control (IRON) group continued on this protocol throughout the experiment, while sham-operated (SHAM) rats were given saline (2.5 mL/kg, SC) injections following surgery.

#### 2.3.2. Presurgical Meal Pattern Monitoring

Rats were moved into the customized FCM home cages and given five 4-oz. jars of standard rodent powdered chow (Figure 2). Food intake was monitored for 22 h each day, starting approximately 5 h after lights went on (±15 min). During the 2-h maintenance period each day, the rats were weighed and vaginal smear samples were taken. All jars and bottles were weighed. Starting on the fifth day, the rats were acclimated the cafeteria diet foods (Table 1). Access was given each day to one jar containing one of the cafeteria diet choices, and a second jar containing powdered rodent chow. These foods were chosen to provide a variety of sugar and fat content and were all preferred by rats during food acclimation days (>80% of total energy intake of each rat was of the cafeteria diet choice when presented alongside powdered chow). During the 2-h maintenance period, jars were weighed and replaced with fresh food. The other food access locations were blocked with overturned empty food jars.

Following food acclimation days, the rats were all given the entire array of the cafeteria diet foods and two bottles of water. Placement of each food was rotated daily to avoid positional bias. The bottles and rats were also weighed as before, and vaginal smear samples were taken. The cafeteria diet was provided in this way for 8 days. After the end of the last pre-surgical test day, rats were returned to standard housing as described for the acclimation period. The rats were weighed daily for one week to monitor for a stable body mass prior to surgical procedures, and to allow reacclimation to the housing space. Estrous cycle sampling was not conducted during this stabilization period or during surgery and recovery phases.

### 2.4. Roux-en-Y Gastric Bypass Surgery and Recovery

Prior to surgery, rats were acclimated for one night to the housing and foods to be used during post-surgical recovery. The post-surgical recovery cage consisted of a standard polycarbonate cage fitted with absorbent untreated cageboard (Techboard, Shepherd Specialty Papers; Watertown, TN, USA) below a raised stainless-steel wire floor insert. The soft recovery foods provided included a thin chow mash (1 part powdered chow to 4 parts water) and a custom-prepared gelatin diet (<1 kcal/g; corn starch, whey powder, corn oil, gelatin, vitamin supplementation, and water; see [60]). On the night prior to surgery, the rats were placed in a clean recovery cage without food but with access to water. The rats were assigned to either the RYGB, SHAM or IRON nonsurgical control group in an attempt to counterbalance pre-surgical body mass, pre-surgical intake of powdered chow and cafeteria diet, and meal size and number for powdered chow (Days 3 & 4) and cafeteria diet (Days 7 & 8).

Aseptic technique was used to prepare materials and to perform the surgery, which is described in detail elsewhere [51]. Briefly, the rat was anesthetized with isoflurane (induction at 5%, maintenance on a nosecone at <3% in 1L O_2_/min). A midline laparotomy exposed the abdominal cavity. The upper jejunum was transected ~10 cm aborally from the ligament of Trietz (roughly where the duodenum meets the jejunum), and each end was ligated to form two stumps. The biliopancreatic limb was made by a side-to-side anastomosis of the jejunal stump oral to the transection line to a portion of the jejunum ~25–28 cm oral to the cecum. The remnant stomach remained continuous with the biliopancreatic limb but was transected ~5 mm aboral to the esophageal junction and closed with suture. The alimentary limb was made by a side-to-side anastomosis of the aboral jejunal stump to the gastric pouch. Sham surgeries were performed by placing suture at the same locations in the gastrointestinal tract, but without transecting tissue. Following each procedure, the abdominal muscles and skin were closed separately with suture. All rats received prophylactic injections of antibiotic (enrofloxacin, 2.3 mg/kg, SC) and analgesic (carprofen, 5 mg/kg, SC) on the day of surgery and for 3 days afterwards.

After recovery from anesthesia, rats were returned to a clean recovery cage and left without food but with access to water. Starting the morning after surgery, rats were given small rations of the soft recovery foods to allow time for the anastomoses to heal. These rations increased in size across days, until the rats were eventually given ad libitum powdered chow and then standard pellets again. RYGB rats returned to pelleted chow by postoperative day 14 (range: 9–14 days). All rats had recovered from RYGB surgery by postoperative day 17. Sham rats and those in the IRON group went through the same food restriction and rationing process as RYGB rats but proceeded through the recovery stages more quickly. They returned to standard chow pellets in seven days.

### 2.5. Post-Surgical Measurements

#### 2.5.1. Post-Surgical Estrous Baseline

Starting five days after the last RYGB rat had recovered and 14 days before post-surgical food monitoring began, daily vaginal swab samples were collected and analyzed as described above. Samples were collected throughout and for four days following the end of Post-surgical Meal Pattern Monitoring.

#### 2.5.2. Post-Surgical Meal Pattern Monitoring

Rats were again moved to the custom cage units of the FCM and given five jars of powdered rodent chow and two bottles of water for four days. After this initial reacclimation period, the cafeteria diet was presented for eight days as described above. Finally, rats were returned to powdered chow (five jars) to assess possible hypophagia resulting from the removal of the cafeteria diet. After four days with powdered chow, the rats were returned to standard housing.

#### 2.5.3. Body Composition Scans

Five to eight days after the rats were returned to standard housing, the animals were transferred to a second facility that houses an EchoMRI (EchoMRI-500™, EchoMRI; Houston, TX, USA). After one night in the new facility, body composition scans were conducted. The animal was briefly restrained in a Plexiglas cylinder, placed into the EchoMRI for 1–3 min, and then returned to the home cage. Fat mass, lean mass, and water mass were determined. The following day, rats were returned to the main housing facility for the duration of the study and were given five days to reacclimate to the facility before starting the protocol for cardiac blood collection.

#### 2.5.4. Postprandial Cardiac Blood Collection and Protein Analysis

Food was removed in preparation for the feeding schedule required for postprandial blood draws. Approximately 23.5 h later, the rats were provided a single jar of powdered rodent chow for 30 min; this was repeated the following day. On the third day, the rats were provided a single jar of sugar/fat whip for 30 min to induce a postprandial gut hormone response to the familiar high-fat/high-sugar food. The jar was weighed to calculate the amount each rat consumed. Thirty min after the end of the feeding period, rats were given a lethal injection of euthanasia agent (0.5 mL of Euthasol, 390 mg/mL sodium pentobarbital, IP). A thoracotomy was performed to expose the heart, and ~3 mL blood was drawn via cardiac puncture from the left ventricle. The blood was collected into EDTA tubes and immediately spun in a 4 °C centrifuge at 2400 rpm for 15 min. The plasma was separated and stored in aliquots at −80 °C until analyses could be completed.

The gut peptide glucagon-like peptide 1 (GLP-1) and the iron-binding protein ferritin were quantified via enzyme-linked immunosorbent assay (ELISA; Millipore Sigma; St. Louis, MO, USA) as functional measures of RYGB and iron supplementation, respectively.

### 2.6. Data Analysis

Of the 17 rats that underwent RYGB surgery, five died of post-surgical complications. One SHAM rat died post-surgically. Their data are not included in any figures or analyses. The final group sizes were: SHAM, *n* = 7; IRON, *n* = 7; RYGB, *n* = 11.

From the meal pattern monitoring phases, the weights recorded every 100 ms were combined into 10-s bins and the mean and standard deviations of weights (in mg) within 10-s bins were calculated and stored for each individual load cell. Feeding bouts were determined by large fluctuations in the registered weights that would indicate activity in the jar. Specifically, minimum criteria to determine when feeding occurred were: (1) changes >0.01 g in the average weight across bins, and (2) a standard deviation >100 mg within the preceding bin. These feeding bouts were compiled into meals using a minimum meal size of 1 kcal and a meal termination criterion of 15. The hardware and meal criteria were determined in preliminary work to validate the FCM and included both quantitative (e.g., pause distribution analyses) and qualitative (e.g., videorecords) methods that are not shown here. Using these criteria, >90% of all intake data to be compiled into meals for each rat during each day of meal pattern monitoring. The meal size (total intake in kcal), duration (in min), and the time (in min) between each meal were also calculated, as well as the amount consumed from each available food option within that meal. The proportion of intake from each food was calculated by dividing the intake of each food option in the meal by the total size of the meal. The quantity consumed of each nutrient source (nonsugar carbohydrates, sugar, protein and fat) was calculated for each meal and for total intake by multiplying consumption of each food by the proportion of energy (in kcal) attributed to that nutrient source within that food (Table 1). The proportion of energy from each nutrient source was calculated by dividing the energy from a nutrient source by total energy intake (in kcal). Meal eating rate was calculated by dividing meal size (in kcal) by meal duration. Satiety ratios were calculated by dividing total energy or energy from each nutrient source by the following intermeal interval. Energy density of meals was calculated by dividing the kilocalories consumed in each meal by the total weight (in grams) of all foods consumed in that meal. Both satiety ratios and energy density were calculated for meals consumed on the last two days of the cafeteria diet access post-surgically (days 15–16).

Total energy intake, proportion of energy from each food source and from each nutrient type (nonsugar carbohydrates, sugar, protein and fat) were compared between groups within phases by mixed two-way ANOVA (group × day). The size, duration, and eating rate for all meals and for meals when lights were on and when lights were off as well as the meal number and intermeal interval were compared between groups within a phase and within groups across phases by mixed two-way ANOVAs. Satiety ratios, energy density, physiological measures and the parameters of first meals were compared via two-sample *t*-tests. Comparisons between groups on individual days and within groups across specific days were analyzed by appropriate *t*-tests. Because the IRON and SHAM groups were not ostensibly or significantly different on any intake, food choice, or meal measure, those groups are combined for these analyses. Meal patterns for food acclimation days are shown but given the rapid change in testing conditions across days, those data are not analyzed. Statistical significance was considered a *p*-value ≤ 0.05.

Because estrus was not a primary variable of this study, there are very few cycles within each phase. When studying the role of the estrous cycle here, the meal patterns and food choices during estrus (the dark period prior to the emergence of estrus in samples combined with the light period following the estrus sample) and during diestrus (the dark period prior to the emergence of diestrus in samples combined with the light period following the diestrus sample) for each rat in the last four days of each cafeteria diet phase were isolated and compared via *t*-test. Not every rat went through an entire estrous cycle during the powdered chow testing days, so no analyses were conducted using meal patterns from those portions of the study.

## 3. Results

### 3.1. Total Intake and Food Choices

**Figure 3:**
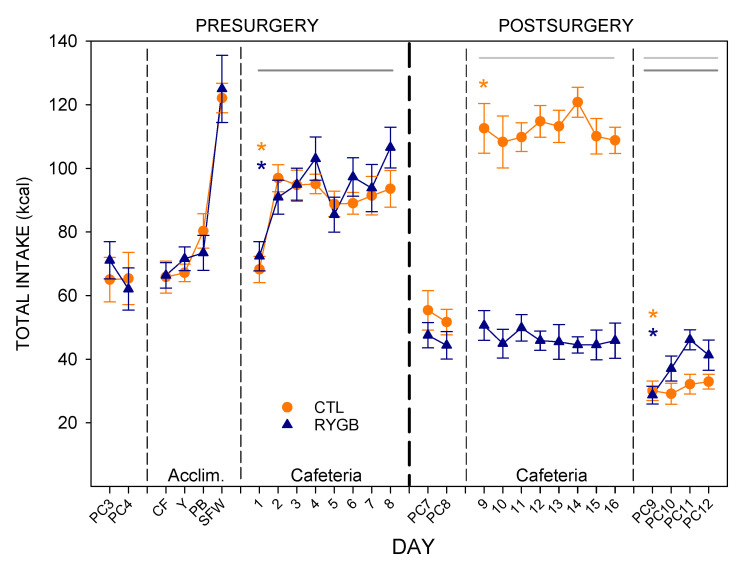
Daily Energy Intake across All 22-h Periods during Meal Pattern Monitoring. Mean (±SE) for CTL rats (combined SHAM and IRON groups, *n* = 14; orange circles) and RYGB (*n* = 11; blue triangles) rats for powdered chow days (PC), acclimation days for each food (Acclim.; CF: chickpea flour; Y: yogurt; PB: peanut butter; SFW: sugar/fat whip), and cafeteria diet days. Dashed vertical lines indicate transitions between diet conditions (thin dashed) and between pre- and postsurgical phases (thick dashed). Statistically significant results from two-way mixed ANOVAs (Table 2) are indicated by horizontal lines: Gray solid (group), dark gray solid (day), or gray dashed (group × day interaction). *: Statistically significant result of a paired *t*-test for the group indicated by color, comparing the marked day to the previous day (Table 3).

**Figure 4:**
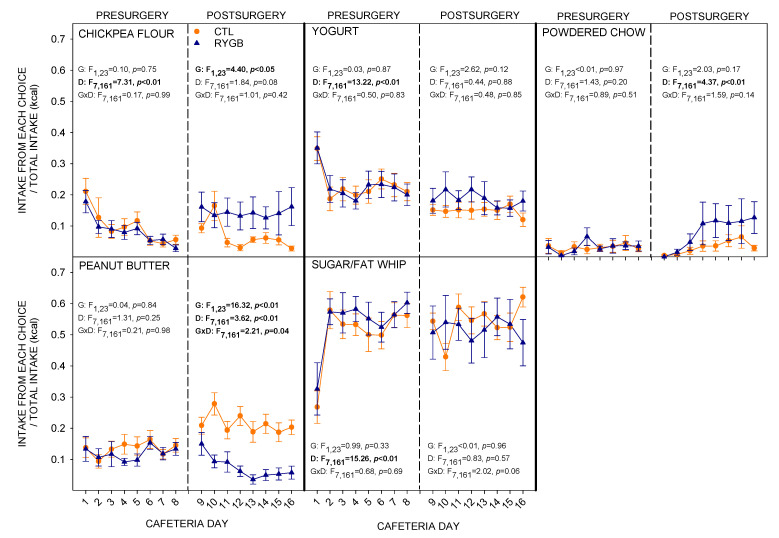
Daily Proportion of Energy from Each Food Choice in the Cafeteria Diet. Mean (±SE) proportion of energy (in kcal) across both pre-surgical and post-surgical days are presented for CTL (combined SHAM and IRON groups, *n* = 14; orange circles) and RYGB (*n* = 11; blue triangles). Inset: results from two-way mixed ANOVAs (G: group, D: day, G × D: group × day), with significant results in bold.

**Figure 5:**
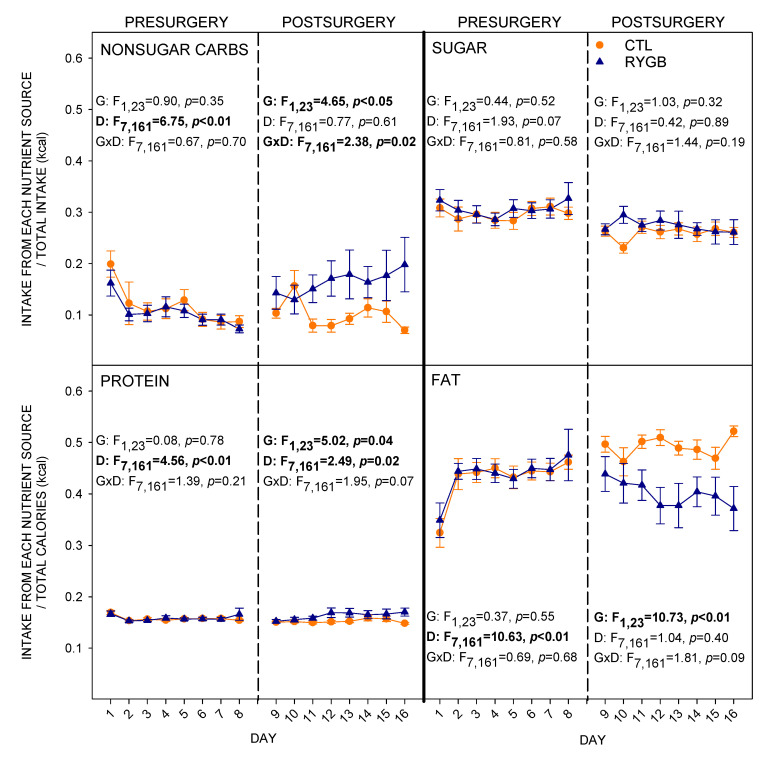
Daily Proportion of Energy from Each Primary Nutrient Source. Mean (±SE) proportion of energy (in kcal) across both pre-surgical and post-surgical days are presented for CTL (combined SHAM and IRON groups, *n* = 14; orange circles) and RYGB (*n* = 11; blue triangles). Inset: results from two-way mixed ANOVAs (G: group, D: day, G × D: group × day), with significant results in bold.

Presurgically, there were no differences between groups in total intake under any diet condition (Figure 3; Table 2). All rats consumed at least 80% of their total energy from the single provided novel food during acclimation days (data not shown). Interestingly, when the animals were moved to the full array of foods in the cafeteria diet, rats actually consumed the least energy on the first day. This was largely driven by the higher preference that day for the yogurt choice, which has the lowest energy density (Figure 4). The rats also consumed the most chickpea flour on the first day of the cafeteria diet, and the least amount of the sugar/fat whip. This profile may result from the fact that the rats had mostly recently had access to the sugar/fat whip whereas other foods had been absent the previous night, an outcome consistent with previous work ([59]). The net effect of these food choices was to cause a significant main effect of day on total energy (Figure 3; Table 2), on proportions consumed of chickpea flour, yogurt, and sugar/fat whip (Figure 4) and on the proportion of nonsugar carbohydrates and fat consumed pre-surgically (Figure 5). Upon return to the FCM after surgery, RYGB and CTL groups consumed similar amounts of powdered chow (Figure 3; Table 2), though the RYGB group tended to consume less energy overall. When presented with the cafeteria diet post-surgically (Cafeteria day 9, Figure 3), CTL animals immediately displayed hyperphagia, consuming almost double the energy than during the last day with powdered chow (PC8), as well as significantly more energy than at the end of the pre-surgical phase (Figure 3; Table 3). However, the RYGB rats did not display the same overconsumption of the cafeteria diet and instead consumed similar number of total energy as they did with the powdered chow, and only about 50% of the energy consumed by the CTL rats (Table 2 and Table 3).

RYGB rats consumed more chickpea flour across the 8-day post-surgery period than did the CTL group, with a main effect of group (Figure 4). RYGB rats also consumed less peanut butter than the CTL rats, with that effect increasing across days in the phase. While RYGB rats tended to consume more powdered chow than CTL rats, both groups slightly increased consumption of powdered chow across the phase, resulting in a main effect of day but no significant result involving group. The post-surgical differences that resulted from these food choice profiles was that RYGB rats consumed more non-sugar carbohydrates (Figure 5), and less fat than CTL rats. There were also small yet significant main effects of group and day on protein consumption. The variability in protein intake across days was low, perhaps reflecting the similar protein content across the cafeteria test foods.

When returned to powdered chow, both RYGB and CTL rats immediately consumed less energy than when on the cafeteria diet, but also significantly less than they did when on powdered chow just nine days previously (Figure 3; Table 2 and Table 3). This hypophagia has been hypothesized to reflect a negative contrast effect, in which the rats are comparing the stimulus properties of their current food (powdered chow) to those of their previous food (cafeteria diet; [83,88]). So, despite not demonstrating increased energy intake when post-surgically switched from powdered chow to the cafeteria diet, the RYGB rats still under-consume the powdered chow when it suddenly becomes the only option available after other foods were removed.

Food choices were also different between the groups post-surgically, with RYGB rats reducing intake of PB and increasing intake of chickpea flour and powdered chow. Individual variation was apparent, with some rats increasing their intake of chickpea flour while others increased their intake of powdered chow, resulting in large standard errors for the RYGB group. The result of these changes was to increase the proportional intake of nonsugar carbohydrates and reduce intake of fat (Figure 5). However, as can clearly be seen in Figure 4 and Figure 5, both groups continued to obtain the majority of their energy intake from foods high in sugar and fat. Thus, while RYGB tends to change their overall preference profile, they remain motivated to consume the high-fat and high-sugar options.

### 3.2. Meal Patterns across the 22-h Period

**Figure 6:**
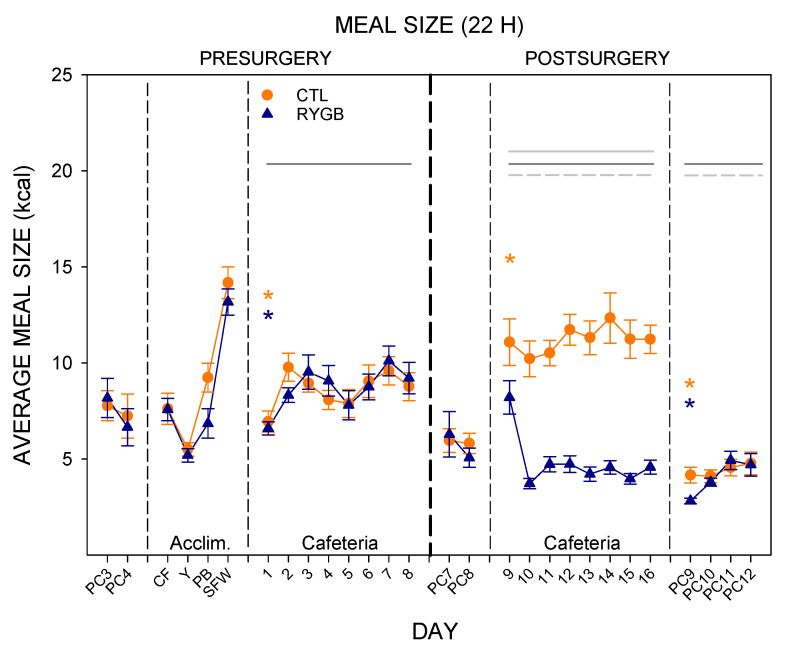
Meal Sizes across All 22-H Periods during Meal Pattern Monitoring. Mean (±SE) for CTL (combined SHAM and IRON groups, *n* = 14; orange circles) and RYGB (*n* = 11; blue triangles) rats for powdered chow days (PC), acclimation days for each food (Acclim.; CF: chickpea flour; Y: yogurt; PB: peanut butter; SFW: sugar/fat whip), and cafeteria diet days. Dashed vertical lines indicate transitions between diet conditions (thin dashed) and between pre- and post-surgical phases (thick dashed). Statistically significant results from two-way mixed ANOVAs (Table 4) are indicated by horizontal lines: Gray solid (group), dark gray solid (day), or gray dashed (group × day interaction). *: Statistically significant result of a paired *t*-test (Table 5) for the group indicated by color, comparing the marked day to the previous day.

Pre-surgically, meal sizes on the cafeteria diet changed significantly across days, with the lowest average meal size occurring on the first day of the cafeteria diet (Figure 6; Table 4 and Table 5). This is perhaps not surprising, given the large quantity of yogurt consumed on the first day (Figure 4), which has the lowest energy density of all the food items provided in this study. Post-surgically, there was a large decrease in meal size for RYGB rats, consuming less than half the energy in each meal compared with CTL animals. There was also a main effect of day, and a group × day interaction (Table 4), likely driven by the fact that RYGB rats consumed their energy in larger meals on the first postsurgical day with cafeteria diet (Cafeteria day 9, Figure 6) than on any other (more on this below).

When the rats were again given powdered chow (PC9–12), both RYGB and CTL groups immediately decreased meal sizes below the average meal size from the last day of the cafeteria diet (Figure 6; Table 5), emulating what was seen with total intake. These rats increased meal sizes again across the final powdered chow days, with a significant effect of day in the two-way ANOVA (Table 4).

**Figure 7:**
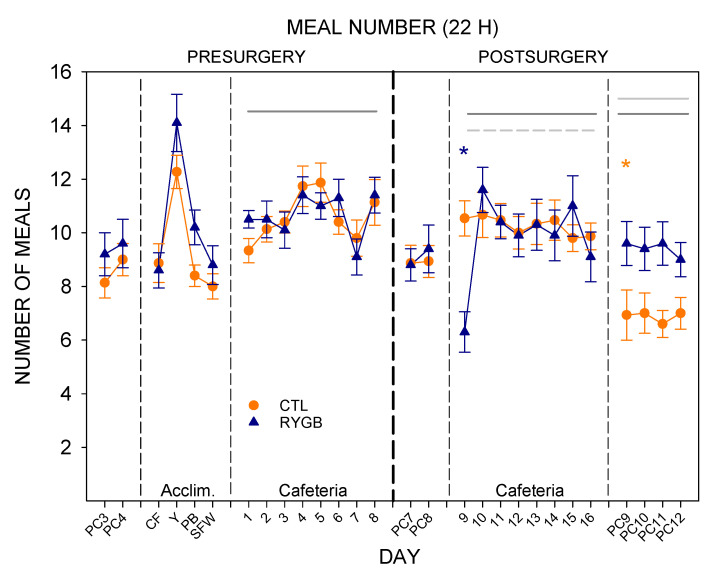
Meal Number across All 22-H Periods during Meal Pattern Monitoring. Mean (±SE) for CTL (combined SHAM and IRON groups, *n* = 14; orange circles) and RYGB (*n* = 11; blue triangles) rats for powdered chow days (PC), acclimation days for each food (Acclim.; CF: chickpea flour; Y: yogurt; PB: peanut butter; SFW: sugar/fat whip), and cafeteria diet days. Dashed vertical lines indicate transitions between diet conditions (thin dashed) and between pre- and post-surgical phases (thick dashed). Statistically significant results from two-way mixed ANOVAs (Table 6) are indicated by horizontal lines: Gray solid (group), dark gray solid (day), or gray dashed (group × day interaction). *: Statistically significant result of a paired *t*-test (Table 7) for the group indicated by color, comparing the marked day to the previous day.

While meal sizes for the cafeteria diet changed dramatically after RYGB, the number of meals initiated was fairly constant (Figure 7). There was no main effect of group for the two-way ANOVA across the post-surgical cafeteria diet days, though there was an effect of day and a group × day interaction caused primarily by a low number of meals for RYGB rats on the first postsurgical day with cafeteria diet (Table 6). These rats initiated fewer meals when on the cafeteria diet than when on powdered chow (Table 7), and also as compared with the CTL rats (Table 6). Meal number increased in the RYGB group on the second post-surgical day with cafeteria diet (Day 10), just as meal size decreased (Figure 6). Interestingly, while meal sizes decreased when the rats were moved back to only powdered chow (PC9), the meal number for RYGB rats remained steady, not differing from the last day of the cafeteria diet (day 16; Figure 7). Thus, except for the first post-surgical day of the cafeteria diet (day 9), the changes in total intake as a result of RYGB were explained by changes in the size of the meals not their frequency.

**Figure 8:**
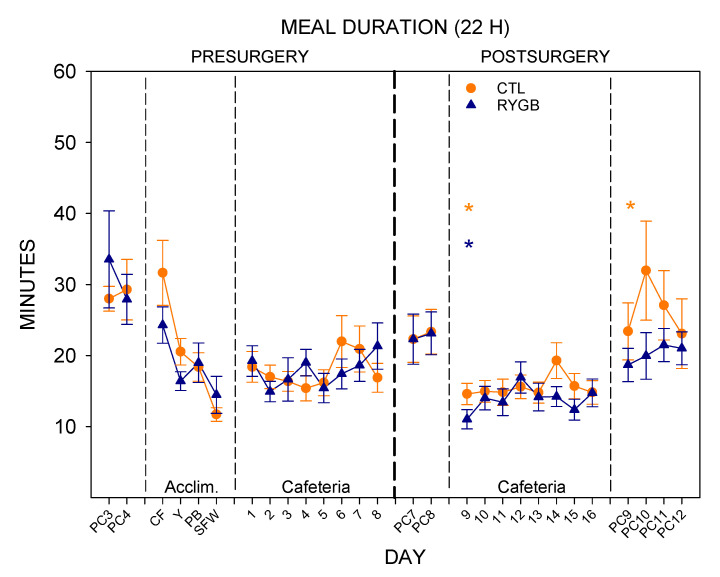
Meal Duration across All 22-H Periods during Meal Pattern Monitoring. Mean (±SE) meal size (in min) for CTL (combined SHAM and IRON groups, *n* = 14; orange circles) and RYGB (*n* = 11; blue triangles) rats for powdered chow days (PC), acclimation days for each food (Acclim.; CF: chickpea flour; Y: yogurt; PB: peanut butter; SFW: sugar/fat whip), and cafeteria diet days. Dashed vertical lines indicate transitions between diet conditions (thin dashed) and between pre- and post-surgical phases (thick dashed). Statistically significant results from two-way mixed ANOVAs (Table 8) are indicated by horizontal lines: Gray solid (group), dark gray solid (day), or gray dashed (group × day interaction). *: Statistically significant result of a paired *t*-test (Table 9) for the group indicated by color, comparing the marked day to the previous day.

Despite a reduction in meal size for RYGB rats, meal duration did not differ between groups (Figure 8; Table 8). Both groups decreased duration slightly when moving from powdered chow to cafeteria diet post-surgically (Table 9).

**Figure 9:**
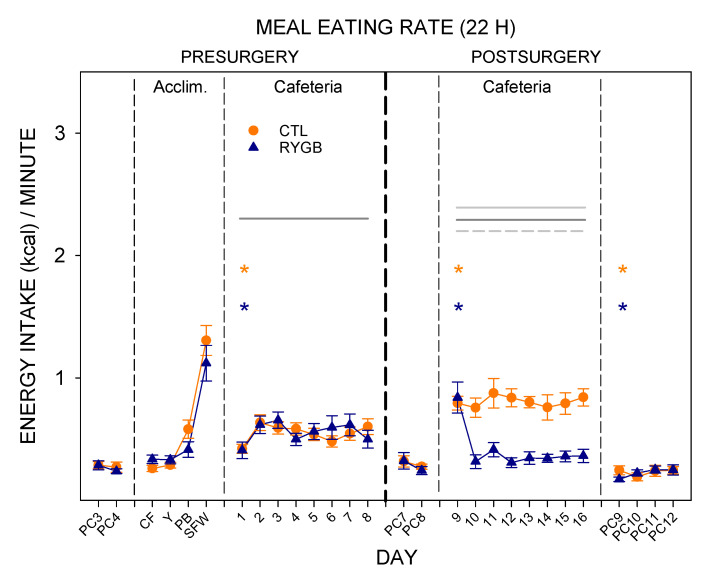
Meal Eating Rate across all 22-H Periods during Meal Pattern Monitoring. Mean (±SE) rate of consumption in kilocalories/minute for CTL (combined SHAM and IRON groups, *n* = 14; orange circles) and RYGB (*n* = 11; blue triangles) rats for powdered chow days (PC), acclimation days for each food (Acclim.; CF: chickpea flour; Y: yogurt; PB: peanut butter; SFW: sugar/fat whip), and cafeteria diet days. Dashed vertical lines indicate transitions between diet conditions (thin dashed) and between pre- and post-surgical phases (thick dashed). Statistically significant results from two-way mixed ANOVAs (Table 10) are indicated by horizontal lines: Gray solid (group), dark gray solid (day), or gray dashed (group × day interaction). *: Statistically significant result of a paired *t*-test (Table 11) for the group indicated by color, comparing the marked day to the previous day.

Given that meal durations did not differ, rate of consumption within a meal (meal eating rate; kcal/min) mirrored meal size (Figure 9). There were no differences between the meal eating rate of the groups pre-surgically or while on powdered chow post-surgically (Table 10). However, RYGB rats consumed their meals at a 2.5-times slower rate across cafeteria diet days, except for Cafeteria day 9 (Table 11).

**Figure 10:**
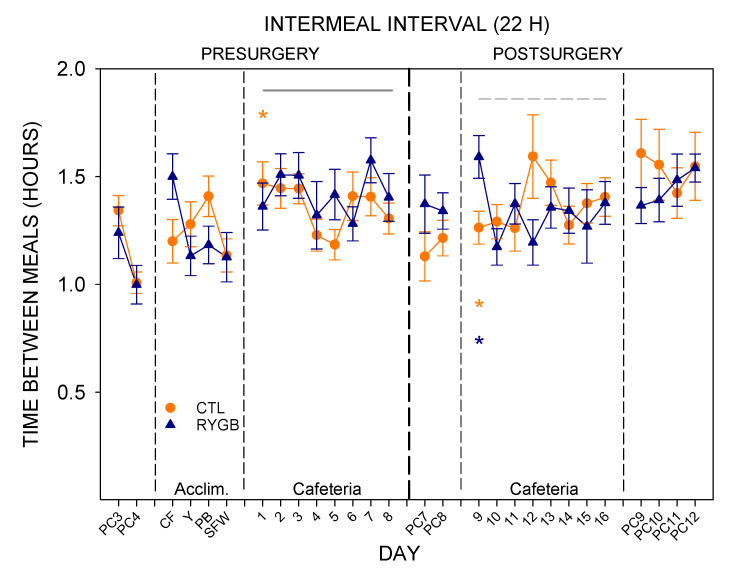
Intermeal Intervals across All 22-H Periods during Meal Pattern Monitoring. Mean (±SE) time between meals (in hours) for CTL (combined SHAM and IRON groups, *n* = 14; orange circles) and RYGB (*n* = 11; blue triangles) rats for powdered chow days (PC), acclimation days for each food (Acclim.; CF: chickpea flour; Y: yogurt; PB: peanut butter; SFW: sugar/fat whip), and cafeteria diet days. Dashed vertical lines indicate transitions between diet conditions (thin dashed) and between pre- and post-surgical phases (thick dashed). Statistically significant results from two-way mixed ANOVAs (Table 12) are indicated by horizontal lines: Gray solid (group), dark gray solid (day), or gray dashed (group × day interaction). *: Statistically significant result of a paired *t*-test (Table 13) for the group indicated by color, comparing the marked day to the previous day.

Like meal number and duration, the length of time between meals (average intermeal interval) was similar between groups (Figure 10; Table 12). Post-surgically, there was a significant group × day interaction (*p* < 0.05) in intermeal interval, likely reflecting the low number of meals by RYGB animals on the first day of cafeteria diet post-surgically (day 9, Figure 10). Both groups increased the intermeal interval in the transition from powdered chow to cafeteria diet pre-surgically and post-surgically (Figure 10; Table 13), which suggests that the cafeteria diet is more satiating than powdered chow.

**Figure 11:**
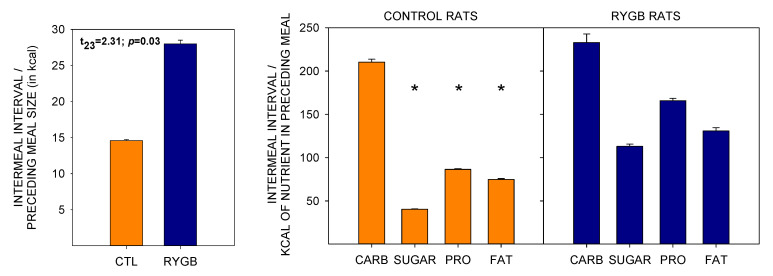
Satiety Ratios in a Meal and from Each Nutrient Type. Mean (±SE) satiety ratios for CTL (orange) and RYGB (blue) groups for meals from the last two days of post-surgical cafeteria diet access (days 15–16), calculated by dividing the intermeal interval (in min) by the total kilocalories in the previous meal (left), or by the kilocalories from a single nutrient type in the previous meal (right). The result of a two-sample *t*-test comparing satiety ratios of a full meal is inset in the left panel. Two-sample *t*-tests between groups were run to compare satiety ratios for each nutrient type. Carbohydrates: t_23_ = 0.98; *p* = 0.09; Sugar: t_23_ = 3.17, *p* < 0.01; Protein: t_23_ = 3.91, *p* < 0.01; Fat: t_23_ = 2.31; *p* = 0.03. *: significant results of two-sample *t*-tests comparing between groups.

**Figure 12:**
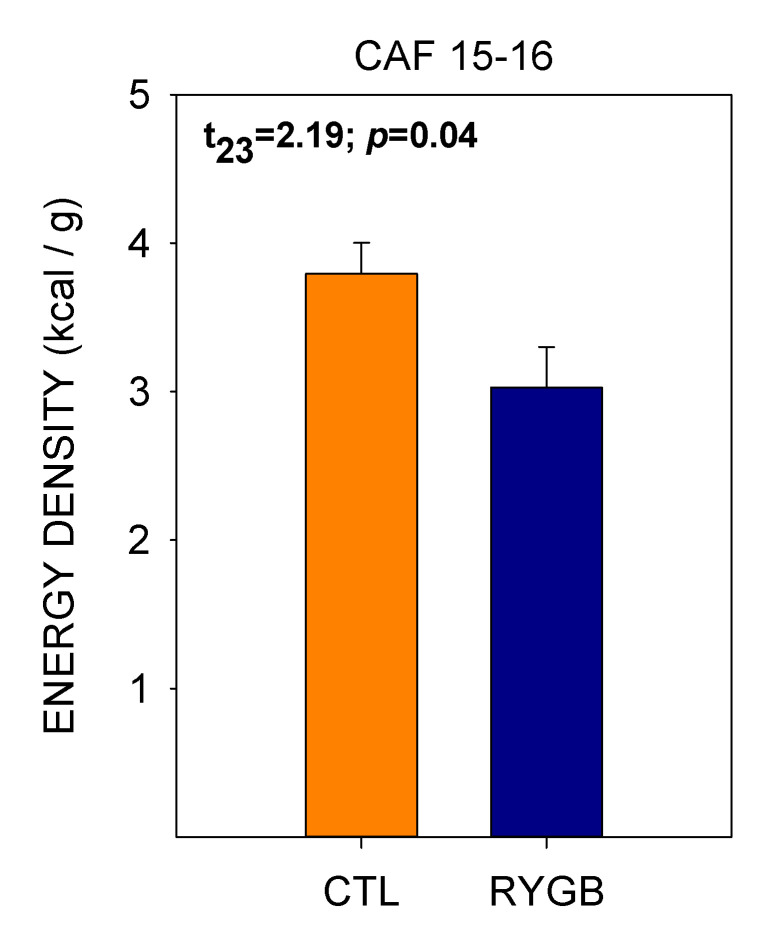
Energy Density of Meals. Mean (±SE) energy density for all meals consumed during the last cafeteria diet days by CTL (*n* = 14; orange bars) and RYGB (*n* = 11; blue bars). Inset: results of *t*-tests comparing groups.

The decreased meal sizes with no change in intermeal interval yielded a satiety ratio (pause after a given meal (min))/(size of that meal (kcal)) in RYGB animals almost double that of CTL rats (Figure 11) by the end of postsurgical cafeteria diet access, meaning that the energy consumed during a meal was more effective at delaying the onset of the following meal in the RYGB group. However, the increase in satiety ratio for RYGB animals did not extend to all nutrient types. While the RYGB satiety ratio was higher for sugars, protein and fat, there was no difference between groups for nonsugar carbohydrates. Also, as RYGB rats altered food choices across days of access to cafeteria diet post-surgically (Figure 4), the average energy density of each meal decreased (Figure 12).

In summary, after the first day on cafeteria diet post-surgically, RYGB rats decreased their average meal size but did not change meal number, ultimately causing a reduction in total energy intake over the 22-h period. The animals also consumed energy more slowly in each meal with generally no change in meal duration or in time between meals. These changes were not found when the animals were given only powdered chow. However, when returned to powdered chow after being maintained on the cafeteria diet, RYGB rats decreased their total intake by further decreasing their meal sizes on the first day of powdered chow (Day 9).

### 3.3. Nycthemeral Patterns of Intake

When the same meal pattern parameters within the light and dark periods are examined separately, it becomes clear that the RYGB animals were sometimes consuming food differently within each period of the day in distinct ways from CTL that were not apparent from only analyzing the meal patterns averaged across the entire 22-h period.

**Figure 13:**
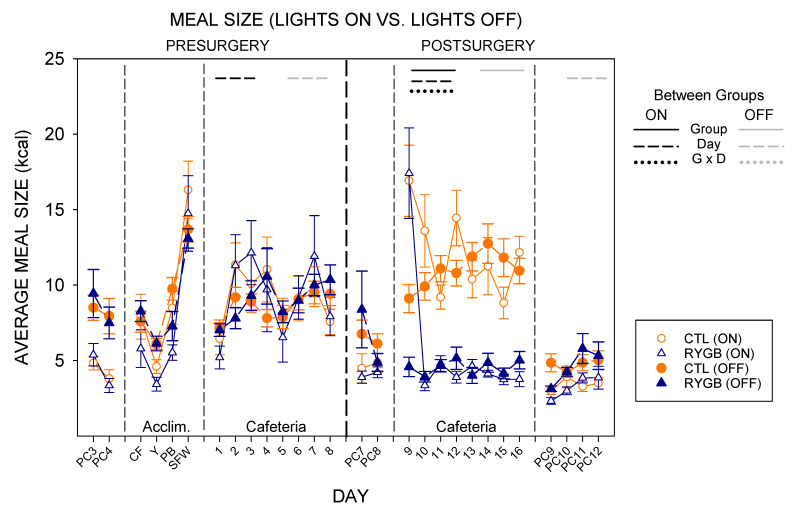
Meal Size for Meals with Lights On vs. Lights Off. Average (±SE) meal sizes when lights were on (open symbols) or off (filled symbols) for each day for CTL (combined SHAM and IRON groups, *n* = 14; orange circles) and RYGB (*n* = 11; blue triangles) rats for powdered chow days (PC), acclimation days for each food (Acclim.; CF: chickpea flour; Y: yogurt; PB: peanut butter; SFW: sugar/fat whip), and cafeteria diet days. Dashed vertical lines indicate transitions between diet conditions (thin dashed) and between pre- and post-surgical phases (thick dashed). Statistically significant results from between-group two-way mixed ANOVAs (Table 14) are indicated by the legend; within-group ANOVA results are found in Table 15.

As expected from prior published work ref or leave to discussion [71,89,90], powdered chow meal sizes while lights were off, when rats are active, are larger than meal sizes while lights were on, when rats are typically dormant (Figure 13). With either subset of meals, there were no group differences pre-surgically for either diet or post-surgically with powdered chow, similar to overall intake (Table 14). While on the cafeteria diet post-surgically, RYGB rats consumed smaller meals in both lights-on and lights-off periods (Figure 13; Table 14), like the results of average meal size across the entire 22-h period (Figure 6). However, there was also a significant effect of day and a group × day interaction for meal sizes when lights were on—with RYGB meal sizes during the lights-on period of the first postsurgical day with cafeteria diet (Cafeteria day 9) being very large compared with any other day (Figure 13; Table 14 and Table 15). In fact, RYGB meals during lights-on hours on the first day post-surgically were very similar to the meal size of CTL rats. By lights-off on that same day, the meal sizes of RYGB rats were already very low compared with the CTL group. Interestingly, access to cafeteria diet tended to abate the difference in meal sizes across the light cycle; there were no differences within surgical condition for lights-on vs. lights-off meal sizes pre-surgically, or for CTL animals post-surgically (Table 15). Meal sizes were different between lights-on and lights-off for RYGB rats while fed the cafeteria diet post-surgically, but this was largely driven by the very large meal size for light-on meals on the first postsurgical cafeteria diet day (day 9). In other studies, access to food choices do tend to increase daytime (light-on) meal sizes substantially [71,91,92,93], so the effect here is consistent. When returned to powdered chow after cafeteria diet, there was no difference in meal sizes for lights-on or lights-off meals, though RYGB animals consumed similar amounts of energy per meal across the light cycle (Table 15).

**Figure 14:**
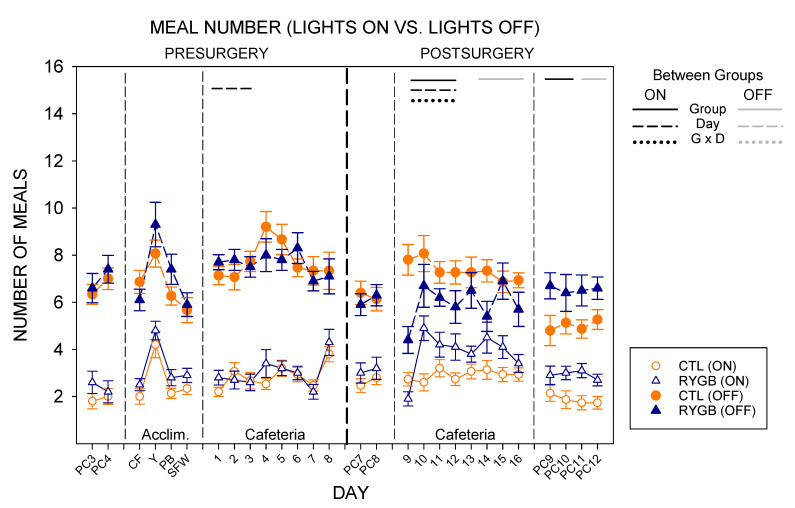
Meal Number for Meals with Lights On vs. Lights Off. Average (±SE) meal number when lights were on (open symbols) or off (filled symbols) for each day for CTL (combined SHAM and IRON groups, *n* = 14; orange circles) and RYGB (*n* = 11; blue triangles) rats for powdered chow days (PC), acclimation days for each food (Acclim.; CF: chickpea flour; Y: yogurt; PB: peanut butter; SFW: sugar/fat whip), and cafeteria diet days. Dashed vertical lines indicate transitions between diet conditions (thin dashed) and between pre- and postsurgical phases (thick dashed). Statistically significant results from between-group two-way mixed ANOVAs (Table 16) are indicated by the legend; within-group ANOVA results are presented in Table 17.

The number of daytime meals were similar between groups pre-surgically (Figure 14). Post-surgically, both groups initiated similar numbers of meals for both lights-on and lights-off hours for powdered chow. On the first day of cafeteria diet post-surgically, both groups initiated similar numbers of lights-on meals, but RYGB rats consumed more meals when lights were on than did CTL rats on the cafeteria diet across the postsurgical period, yielding a significant effect of group, day, and an interaction in two-way ANOVAs (Table 16). RYGB rats also tended to initiate fewer meals during the dark period. Nevertheless, RYGB rats initiated more meals during lights-off than during lights-on hours, a pattern similar to the CTL group (Figure 14; Table 17).

However, because the RYGB animals had greater number of meals during the lights-on phase than CTL rats and a lesser number during the lights-off phase, the nycthemeral disparity in meal number was less in the RYGB group. Indeed, the ratio of lights-on meal number to lights-off meal number averaged across the eight postsurgical days with cafeteria diet is significantly higher in the RYGB compared with CTL group (Nycthemeral Meal Number Ratio: CTL = 0.43 ± 0.02; RYGB = 0.76 ± 0.06, *p* = 0.02). When returned to powdered chow, RYGB animals initiated more meals than CTL animals in both light-cycle phases, despite both groups still showing a typical pattern with more meals during the dark period.

**Figure 15:**
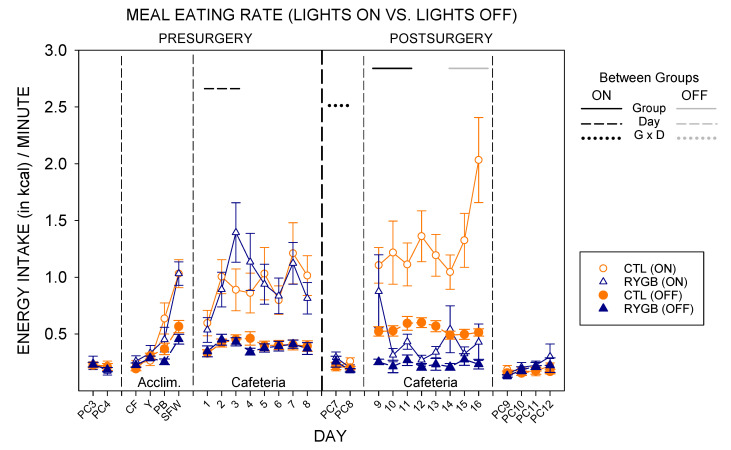
Meal Eating Rate for Meals with Lights On vs. Lights Off. Mean (±SE) rate of consumption in kilocalories/minute when lights were on (open symbols) or off (filled symbols) for each day for CTL (combined SHAM and IRON groups, *n* = 14; orange circles) and RYGB (*n* = 11; blue triangles) rats for powdered chow days (PC), acclimation days for each food (Acclim.; CF: chickpea flour; Y: yogurt; PB: peanut butter; SFW: sugar/fat whip), and cafeteria diet days. Dashed vertical lines indicate transitions between diet conditions (thin dashed) and between pre- and post-surgical phases (thick dashed). Statistically significant results from between-group two-way mixed ANOVAs (Table 18) are indicated by the legend; within-group ANOVA results are presented in Table 19.

While meal eating rates (kcal/min in a meal) are similar between lights-on and lights-off meals on powdered chow, both groups tended to increase the rate of consumption for meals when lights were on, relative to lights-off meals, when provided the cafeteria diet (Figure 15). Post-surgically, RYGB rats consumed the cafeteria diet at a slower rate post surgically than CTL animals in both parts of the light cycle (Table 18). CTL animals continued to demonstrate a higher rate of consumption during lights-on meals than when lights were off post-surgically, as was seen in all animals pre-surgically (Table 19). RYGB animals also consumed kilocalories at a higher rate during meals initiated while the lights were on, driven primarily by the high rate on the first postsurgical day (Figure 15; Table 19). The difference between groups was primarily seen during cafeteria diet exposure, with no main effect of group for any phase of powdered chow.

**Figure 16:**
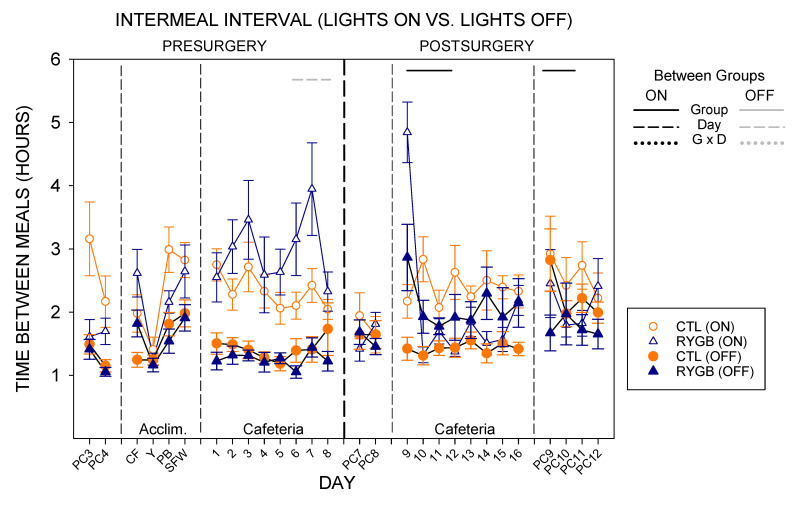
Intermeal Interval for Meals with Lights On vs. Lights Off. Mean (±SE) intermeal interval in hours when lights were on (open symbols) or off (filled symbols) for each day for CTL (combined SHAM and IRON groups, *n* = 14; orange circles) and RYGB (*n* = 11; blue triangles) rats for powdered chow days (PC), acclimation days for each food (Acclim.; CF: chickpea flour; Y: yogurt; PB: peanut butter; SFW: sugar/fat whip), and cafeteria diet days. Dashed vertical lines indicate transitions between diet conditions (thin dashed) and between pre- and post-surgical phases (thick dashed). Statistically significant results from between-group two-way mixed ANOVAs (Table 20) are indicated by the legend; within-group ANOVA results are presented in Table 21.

The intermeal interval between cafeteria diet meals also changed for RYGB rats compared with the CTL group, in general (Figure 16). There were no differences between groups in intermeal interval before surgery. After surgery, intermeal intervals were still similar between groups for powdered chow days during both lights-on and lights-off meals (Table 20). On cafeteria diet, there was a significant interaction (group × day) for pauses between lights-on meals with RYGB rats taking shorter breaks between meals except for the first postsurgical day (Cafeteria day 9), and a main effect of group for lights-off meals with RYGB rats taking longer breaks between meals. Overall, RYGB rats had similar intermeal intervals between lights-on and lights-off meals even with a significant interaction (lights × day), likely due to the very large intermeal interval for the lights-on period of the first day of cafeteria diet postsurgically (day 9; Table 21); CTL animals took significantly longer pauses after lights-on meals than after lights-off meals when on the cafeteria diet, as did all animals pre-surgically (Table 21). Taken altogether, the meal pattern that emerges for RYGB animals is one that elicits a more consistent pace of consumption across the entire 22-h period: evenly-sized small meals (Figure 13), eaten at a consistent rate (Figure 15), almost evenly spaced across the entire day (Figure 14 and Figure 16).

The meal patterns during the first day of cafeteria diet access stands out from the other days, particularly the meals initiated during the lights-on period. Consideration of the logistics of the study may provide some help in understanding why that might have occurred. As the session starts each day while lights are on, the daytime hours are the first five hours of each 22-h monitoring period, and the last five hours. In general, it was observed that rats did not initiate many meals in the final five hours of each day; meaning, most of the intake from the “daytime”, when lights are on, was typically within the first five hours. In addition, rats tended to initiate a meal almost immediately when access to the cafeteria diets was restored each day. It was observed that every rat in this study initiated the first meal on within 1 min of the start of the 22-h period on the first day of cafeteria diet post-surgically.

**Figure 17:**
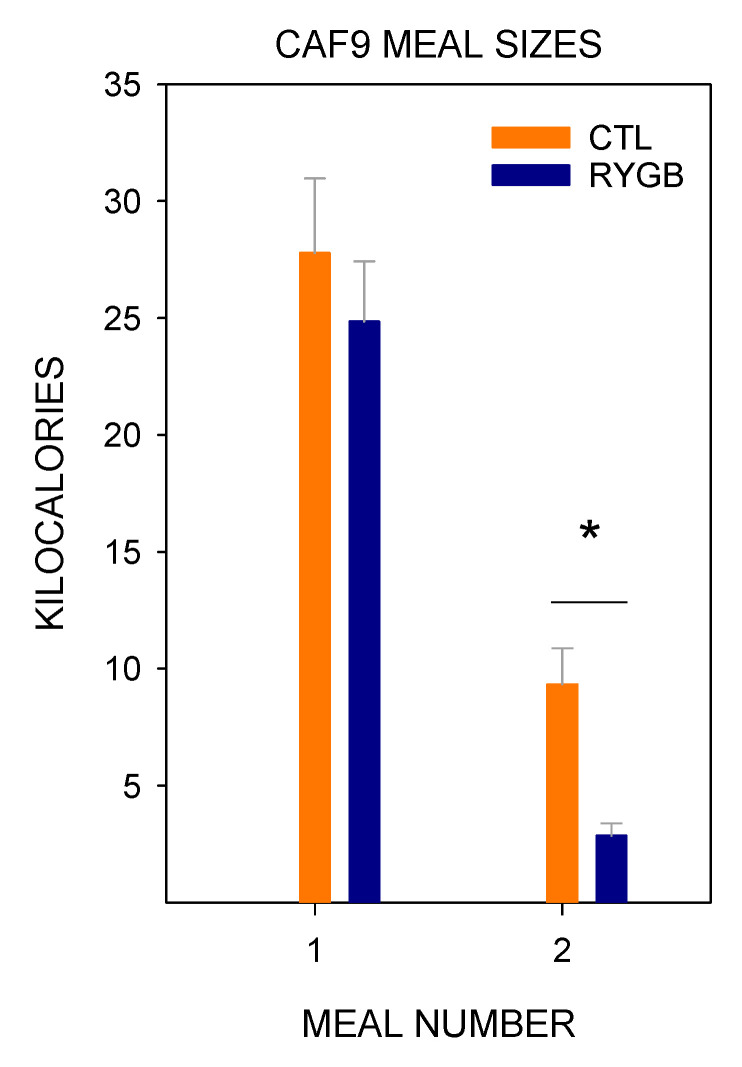
Energy Consumed in the First and Second Meal of Cafeteria Diet Postsurgically. Mean (±SE) meal size (in kcal) for Control (CTL; combined SHAM and IRON groups, *n* = 14) and RYGB (*n* = 11) rats. *: Significant results (*p* < 0.05) from two-sample *t*-tests.

**Figure 18:**
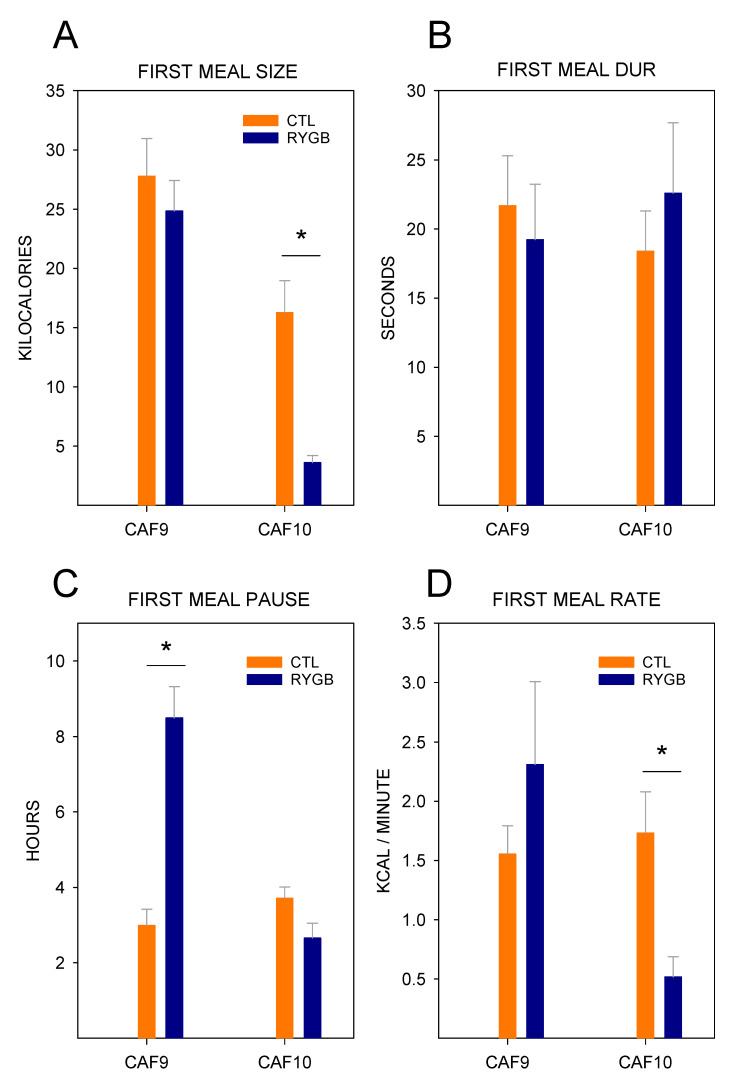
Meal Patterns for the First Meal Consumed on the First Day of Cafeteria Diet Exposure Postsurgically (CAF9) and the Second Day (CAF10). Mean (±SE) size (**A**), duration (**B**), postmeal pause (**C**) and first meal rate (**D**) for CTL (combined SHAM and IRON groups, *n* = 14) and RYGB (*n* = 11) rats. *: Significant results (*p* ≤ 0.05) from two-sample *t*-tests.

In fact, the very first meal dominated the daytime intake on that day. Both groups consumed a very large first meal (Figure 17), with no significant difference in energy intake (t_23_ = 0.18, *p* = 0.86). By the second meal, however, while both groups significantly reduced the size of the meal, RYGB rats were already consuming less energy per meal than CTL animals (t_23_ = 3.69, *p* < 0.01). This very large first meal was not repeated at the start of the second postsurgical day with the cafeteria diet or ever again (Figure 18); on Caf10, the size of the first meal was significantly smaller for RYGB rats than CTL animals (t_23_ = 3.95, *p* = 0.79). Not only did RYGB rats consume as many kilocalories as CTL animals in their first postsurgical meal of cafeteria diet, but they also consumed it in a similar amount of time (t_23_ = 0.28, *p* < 0.01), leading to a consumption rate that was similar to CTL animals, as well (t_23_ = 0.97, *p* = 0.35). However, after this large meal, RYGB rats waited much longer to consume a second meal, as the intermeal interval was over twice as long for RYGB rats as that for CTL animals (t_23_=5.70, *p* < 0.01). As with meal size, by the second day of cafeteria diet post-surgically (Caf10), the pattern of the first meal now looked similar to the overall results: RYGB rats consumed a smaller meal in the same amount of time as CTL animals (t_23_ = 0.41, *p* = 0.69), resulting in a slower consumption rate (t_23_ = 3.40, *p* < 0.01). Thus, it seems that RYGB animals will avidly consume the cafeteria diet, but after only one meal learned to adjust their meal patterns to compensate for the effects of the surgery.

Along with differences in meal patterns depending on whether lights were on or off, there were also slight differences in the content of those meals. On the first postsurgical exposure to cafeteria diet (CAF-9), rats in either group tended to consume a lower proportion of energy from complex carbohydrates, less protein, and more fat in meals initiated when lights were on or for meals across the entire 22-h period (Figure S1; Table S1). There were no group differences, or differences in lights-off meals compared with all meals for that day. By the final day of cafeteria diet, some group differences had emerged when comparing subsets of meals. By the end of cafeteria diet testing (CAF-16, Figure S1), the CTL group consumed proportionally more nonsugar carbohydrates during the lights-on period compared with when lights were off, and RYGB animals consumed proportionally less. Energy from fat was still higher in RYGB animals when lights were on compared to when lights were off, but CTL animals were consuming slightly less proportions of fat during the day (Figure S1; Table S1). Overall, though, the differences in nutrient content between subsets of meals in the CTL rats were fairly minor, indicating that these animals maintained a moderately uniform control over what they consumed across the 22-h period.

### 3.4. Drinking Behavior

There was no difference between RYGB and CTL groups for water consumed post-surgically (Figure S2). RYGB rats licked at a slightly slower rate during cafeteria diet testing, with a significantly slower interlick interval.

### 3.5. Estrous Cycle

Given the reported changes in meal patterns due to estrus (e.g., [94,95]), and the possible interactions between estrus and the changes in gut hormones after RYGB surgery [96,97,98], estrous cycle samples were taken daily throughout the study, except for the surgical and immediate postoperative periods. With the duration of each phase of the study being so short relative to estrous cycling, it was not possible to analyze the effect of estrus on meal patterns rigorously. Average estrous cycle duration was similar between groups post-surgically (RYGB: 4.2 days (±0.1 day); CTL: 4.3 days (±0.2 days)). To compare across days and to minimize the effect of changes in diet intake across the phase of the study, only data from the end of each cafeteria diet phase were considered in the analysis. The last day of estrus during presurgical and postsurgical cafeteria diet exposure for each animal was compared with that animal’s intake and meal patterns for the diestrus phase. This means that for some animals the sample for estrus was earlier in the diet exposure phase than it was for diestrus, and for some animals it was later in the diet exposure. As reported elsewhere, estrus reduced meal sizes of the cafeteria diet for all groups pre-surgically (Figure S3). However, there was also a tendency for rats to consume more meals on the cafeteria diet. The net result is that there was no significant reduction in total energy intake due to estrus, as is typically reported in the literature. After RYGB surgery, there was no longer an effect of estrus on meal size and number, or total intake. It is possible that this is due to a floor effect, as RYGB rats are already consuming small meals. Estrus also did not affect food selection in any group pre- or post-surgically (Figure S4; Table S2), with no significant change in proportion of energy from any food source or nutrient type (Figure S5; Table S2).

### 3.6. Other Physiological Measures

Body mass was similar across presurgical testing, and the groups gained a similar amount of body mass across the meal pattern monitoring period (Figure S6; Table S3). RYGB rats were significantly lower in body mass at the start of postsurgical testing and were stable with no significant change across days in a repeated measure one-way ANOVA (F_15,150_ = 1.689, *p* = 0.08). In contrast, CTL animals not only weighed more at the start of postsurgical testing, but these animals also gained a significant amount of weight across the 16-day postsurgical monitoring period (F_15,205_ = 118.48, *p* < 0.01). As expected, RYGB rats also had a lower percentage of body fat compared with CTL animals.

RYGB rats also had a higher postprandial GLP-1 response than both SHAM and IRON rats after the terminal meal (Figure S7; Table S4). Interestingly, the IRON group also had a higher GLP-1 response than the SHAM group, although there was overlap in individual values between those two groups. A correlation has been reported between levels of ferritin and GLP-1 in plasma ([99]), so it may be that there is some currently unexplained link between iron metabolism and the actions of GLP-1. As expected, IRON rats also had a significantly higher level of plasma ferritin. However, despite receiving weekly doses of iron, RYGB rats had levels of ferritin that were similar to SHAM rats, and significantly less than the IRON group.

## 4. Discussion

### 4.1. Roux-en-Y Gastric Bypass Changes Meal Patterns Primarily by Reducing the Size of Meals

This study is the first to analyze meal patterns in rats provided a complex, multi-food diet after bariatric surgery, representing an important translational step in understanding how RYGB affects feeding behaviors. The most significant change seen in meal patterns for the cafeteria diet was that RYGB surgery reduced energy consumed from a cafeteria diet, primarily by lowering the amount of food consumed in each meal. This is consistent with other findings in both patients and rodents [62,77,78,80,100,101,102]. Given that meal-related behaviors are altered with the addition of multiple food choices (e.g., [81,82,83,84]), it was not altogether clear whether RYGB would substantially change how rats consume food under these conditions; such a study was not possible without a device such as the FCM. The work presented here confirms, for at least the rat model, the primacy of processes responsible for meal termination as opposed to meal initiation in the intake-reducing effects of RYGB, even in the face of multiple choices from palatable diets varying in their sugar and fat content.

Rats also progressively decreased energy taken from fat and increased energy taken from nonsugar carbohydrates, as shown previously with intake measures [59], effectively reducing the energy density of their meals. There are several reasons why the physical changes after the surgery might encourage a reduction in total intake, and that of fat and/or sugar in particular. With elevated levels of satiety hormones like GLP-1, PYY and potentially CCK [35,103,104,105], it would make sense that meals would be smaller. Enhanced satiation and satiety are often cited as a large component of the success of bariatric surgeries [106,107,108]. The high satiety ratios—comparing the delay to the next meal relative to the size of the previous meal—for RYGB animals in this study and others [77,79] are consistent with that hypothesis, as well. However, in intact animals, alterations in prandial endocrine responses are typically counteracted by compensatory mechanisms that result in little total decrease in intake [31,109,110,111,112], so other factors must also contribute after RYGB surgery to maintain overall reductions in calorie consumption [108]. Food intake might also decrease in RYGB animals in part as a function of the intake-reducing effects of jejunal contact with fat. It has been shown that fat infused into the jejunum reduces food intake, and that this process relies on GLP-1 signaling [113,114,115]. When RYGB animals eat, food is deposited almost directly into the jejunum and GLP-1 signaling is elevated [35,51,103,116], making this event a possible candidate for reduced intake when RYGB rats are eating a high-fat diet.

There is also some evidence from patient reports that there are negative visceral consequences of consuming high amounts of sugar and fat. A fast change in blood sugar can trigger symptoms such as dizziness, nausea, vomiting, and diarrhea, namely dumping syndrome [19,20,117,118,119,120]; there are also reports of hyperinsulinemia after the surgery [121]. Malaise is also reported by patients administered high acute doses of satiety hormones as a nonsurgical treatment for obesity, and so the consumption of a meal that triggers a large postprandial response after RYGB could result in similar negative consequences [33,122]. This sort of physiological response has been suggested as an explanation for both patients and rats eating meals that consist of lower energy density after bariatric surgery, as was found here (Figure 11; [12,18,61,77]). It may be, then, that animals learn to reduce intake of particular foods in order to avoid discomfort.

It has been shown in several different contexts that meal size is heavily influenced by the post-ingestive consequences of the food and fluid consumed as well as prior experience with similar items. Rats readily learn to associate the tastes of foods and fluids with the results of their ingestion. Animals can be conditioned to avoid a particular food of fluid after negative feedback such as visceral malaise (illness), or to consume more as a result of a positively reinforcing experience such as with appetition or the correction of a nutritional deficiency [67,123,124,125,126,127,128]. It has also been demonstrated directly that RYGB animals can use postoral cues to modify intake. In one study, rats were given saccharin, a noncaloric sweetener, to drink, followed by an intestinal infusion of corn oil via oral gavage. In subsequent tests, RYGB rats avoided saccharin, while sham-operated rats continued to prefer the saccharin over water [18].

Even when the animal willingly consumes a large amount, that amount can also be influenced by learning and experience. When feedback from the gastrointestinal system is minimized via sham feeding, in which a liquid energy source drains from the stomach via fistula as it is consumed, rats initially drink a similar amount of the fluid as when it normally accumulates in the stomach. It is only with additional sham feeding tests that intake increases [129]. So it is not surprising that rats in this study required some experience with different foodstuffs before behavior changed. It is quite remarkable, though, that these rats only required one meal with the cafeteria diet to modify their feeding behaviors. In the first meal after surgery, all rats consumed a very large meal at a similar rate regardless of group. After this meal, RYGB animals did not eat again for a long time compared with CTL animals and after resuming food consumption their meals were smaller. This finding is similar to other studies wherein RYGB rats initially responded similarly as control animals in other contexts, such as short- and long-term drinking tests, only to reduce intake in subsequent exposures (e.g., [55,59,60,64,130]).

Other studies of meal patterns after RYGB did not report a large first meal. In those studies, though, the animals were also tested with foods or fluids that had been provided during postsurgical recovery. In the present study, rats were not given any diets similar to the cafeteria diet foods during the recovery period, and were only provided a gelatin-based diet that was similar in proportional macronutrient content as the standard rodent chow but with lower energy density. As such, they did not have the opportunity to learn about the consequences of consuming anything that might generalize to the test foods. Indeed, the opportunity to quantify the very first meal consumed when high-fat and high-sugar options were present is precisely why the animals in this study were not maintained on a high-fat diet prior to our experiment to increase body fat further. Indeed, maintenance on a high-fat diet can change the pattern of ingestive behavior toward palatable stimuli in rodent models independently of body fat mass [131].

### 4.2. While Meal Patterns Changed Quickly, the Changes in Food Choices Occur More Slowly across Several Days

The slow adjustment in food choices could be because there were multiple options available, and it took time for animals to associate the new post-ingestive consequences with a particular food. When provided with a single sugar solution, RYGB rats decreased intake after only one exposure to a sugar solution [60], but one behavioral strategy that rats seem to use when presented with several choices is to eat meals that each consist of few food items. This behavior has been suggested to have evolved to allow rats to determine a source of a critical element in which they are deficient or to allow them to better determine the source of any malaise that develops, given that rats cannot vomit to rid themselves of toxic substances they ingest [67,128].

Of course, other processes could contribute to the changes in food choices seen in this study. It cannot be ruled out that some physiological adaptations may have occurred over time as a result of the surgery, and that behavior changed as a result of such adaptations rather than as the result of a learning process [108,116]. One of the most difficult distinctions to make is whether changes in behavior result from the experience with the stimulus directly (learning) or as a secondary effect of physiological changes. More precise studies targeting adaptive processes would need to be run to better understand whether adaptive processes contributed to the results of this study. However, the fact that meal size shows such a rapid modulation in RYGB rats after the return to the cafeteria diet, strongly favors the involvement of a rapid learning process.

### 4.3. Despite Decreasing Their Relative Fat Intake, RYGB Rats Were Still Motivated to Consume a Large Portion of Energy from Fat and Sugar

Another possibility for changes in food choices of RYGB rats is that these animals are no longer motivated to consume the cafeteria diet foods. It has been hypothesized that changes in central circuits as a result of bariatric surgery serve to reduce hedonic eating—eating for the rewarding properties of the foods available rather than for need [66,132,133,134]. Consistent with this, RYGB rats in this study did not significantly increase average meal size nor total intake when post-surgically moved from the powdered chow to the cafeteria diet. However, RYGB rats avidly consumed the cafeteria diet upon first re-exposure to the foods after surgery, suggesting that the lack of hyperphagia during the postsurgical cafeteria diet testing was not directly caused by altered neural circuitry after the surgery. And even after the first meal, when meal sizes returned to the values observed with powdered chow alone, those animals still consumed most of their energy from fat and sugar, suggesting that the cafeteria diet was still found palatable by these animals after surgery. It is true that the within-meal eating rate, which has been considered in other work to be a measure of palatability [135,136], was reduced after the first meal. Thus, a slower rate of intake in meals by RYGB animals would imply that the rats are less motivated to consume the cafeteria diet foods. Eating rate, however, can also be affected by the physical qualities of the foods and any physical manipulations that might limit the ability of an animal to eat such as disruptions in salivation, motor capabilities, and importantly, alterations in gut anatomy such as bariatric surgery [82,137,138,139,140]. Further, in meal pattern analysis the overall eating rate is the amount consumed across the entire meal, but there are likely pauses between bursts of feeding behavior within the meal [141,142]. It may be that microstructural analysis of eating within meals during cafeteria diet exposure would demonstrate that RYGB animals will avidly consume the foods during bursts within the meal, but adjust their within-meal feeding behaviors to include longer pauses between bursts. Unfortunately, the 10-s bins utilized in this study make it difficult to conduct microstructural analyses of these data or to definitively conclude that rate decreased as a result of a change in the motivating orosensory properties of the foods.

The meal patterns of RYGB animals when presented powdered chow after access to the cafeteria diet phase ended also suggest the surgery did not alter the palatability of cafeteria food options as a whole. All rats, regardless of group, significantly reduced their total intake and meal size of the powdered chow compared with the days before when the cafeteria diet was available. In other studies, similar changes have been demonstrated when food choices are removed. The switch in foods results not only in reduced intake and meal sizes, but increased locomotor searching behaviors as if the animals are seeking the “missing” foods [83,88,143,144]. This seems to be related to a negative contrast effect, wherein the stimulus properties of the current (powdered chow) and prior foods (cafeteria diet) are being compared and evaluated, and is not easily explained by any compensatory mechanism to induce weight loss after removal of the food options with the highest energy density [83]. That is, the powdered chow is seemingly less motivating than it was before exposure to the avidly consumed cafeteria diet [145] even for the RYGB rats in this study that did not consume large amounts at high rates.

When considered in context with other results, the reduced eating rate in the RYGB rats may therefore be part of a behavioral strategy to more evenly distribute intake across the entire 22-h period [77,79]. The RYGB rats ate relatively evenly-sized small meals, eaten at a more consistent rate, almost evenly spaced across the entire day. This pattern may help alleviate negative consequences of the ingestion of high-energy foods that the animals are still motivated to consume.

### 4.4. Study Limitations

While these results are generally concordant with previous work, there are some potential limitations. The results here may not generalize to male rats, though the changes in total intake and macronutrient choices are similar to those found in an intake study done with male rats provided similar food items [59]. It also remains possible that the results described here would be different using other human foodstuffs or if testing occurred with foods that vary in nutrient sources but that are more consistent in other characteristics. The foods included here were chosen because (a) they vary in all of the characteristics of foodstuffs available to patients every day, (b) are readily accepted by rats, (c) did not ostensibly spoil over the 22-h period, and (d) were not hoarded.

While there is a general consensus that RYGB reduces fat and sugar intake in rodents, changes in macronutrient selection by humans is less clear [26,29,59,77,146]. There are potential reasons why outcomes from the rodent studies do not mimic the results of direct measures of human food choices after bariatric surgery. First, there are some differences between patient outcomes and rodent models in physiological responses to the surgery. Rodent studies sometimes report changes in energy expenditure (metabolism) that are not always identified in patients [62,147,148,149,150]. In addition, the reported constitutive increase in GLP-1 and PYY seen in rodents are not always observed in patients (see [151] for review). While it is not clear if and how these possible physiological differences would explain why rodents selectively reduce intake of high-energy items while humans do not, they cannot be dismissed.

Second, it is possible, even likely, that the factors that make a rodent model so appealing are contributing to the dissimilarities in behavioral outcomes. While rats have little experience with these food items to allow careful quantification of feeding behaviors, patients have a lifetime of familiarity with a wide variety of foodstuffs. Years of experience consuming diverse foods may have an influence on the capacity of patients to adjust their feeding behaviors through learning. Moreover, while the bariatric intervention is performed in humans with a high level of obesity, there is not a standard definition of obesity in rodents. It is possible that the level of pre-surgical fat mass would affect the changes that result from RYGB surgery observed in this study. As noted above (Section 4.1), we intentionally refrained from maintaining rats on a high-fat diet in the weeks prior to the experimental manipulations because this would have introduced interpretive complications. Also, with regard to rodent work not aligning perfectly with results in patients, while most rodent work is done with single-housed animals to better measure behavior of each individual, eating is, for humans, a social behavior which can ultimately influence what and how much is eaten in a meal [152,153,154]. Finally, the psychological/cognitive factors associated with eating also contribute to how individuals respond to RYGB, and is one of the reasons that patients receive nutritional counseling after the surgery [155,156,157]. There are several ways that human psychological states can impact feeding behaviors. The most notable are eating disorders, which are somewhat common in bariatric candidates and postsurgical patients [158,159]. Some humans also exhibit emotional eating—eating as a comforting response to a heightened state of stress, sadness, or anxiety. This behavioral response has also been correlated with weight regain after bariatric surgery [62,152,158].

### 4.5. Concluding Remarks

Studying behaviors related to a procedure as complex as RYGB is difficult because there are so many variables involved. Despite the imperfect concordance in results between the rodent and human studies, the rodent model gives us critical insight into the fundamental processes controlling intake and selection of foods unencumbered by the cognitive, social, and economic factors associated with human food consumption. This simplification has been essential to understanding the long-term success of bariatric surgery. The work presented here extends previous research by combining meal pattern analysis with a cafeteria diet paradigm, allowing for the observation and quantification of how food choices from a complex array of foodstuffs impact meal patterns. Future studies using this methodology will ultimately help us to better understand the complex interactions between feeding behaviors and the physiological, endocrine, and neural factors involved in the control of food intake.

## Figures and Tables

**Table 1:** Cafeteria Diet Food Choices.

FOODS	DIET TYPE ^1^	KCAL/G	% CARB ^2^	% SUG ^2^	% PRO ^2^	% FAT ^2^
Powdered rodent chow	LS/LF	3.34	57.9	8.9	28.7	13.4
Chickpea flour	LS/LF	3.92	71.5	3.4	17.0	11.5
Low-fat Yogurt	HS/LF	0.79	67.0	65.0	19.7	13.3
Creamy peanut butter	LS/HF	6.38	15.7	5.9	13.7	70.6
Sugar/Fat Whip	HS/HF	5.80	29.2	27.5	13.5	57.3

^1^: Foods presented are categorized as being low (L) or high (H) in sugar (S) and fat (F) relative to other food choices. ^2^: % of total kilocalories that comes from each macronutrient and sugar.

**Table 2:** Two-way ANOVAs Comparing Total Energy Intake between Groups for Each Diet Condition.

**PRESURGERY**	**GROUP**	**DAY**	**GROUP × DAY**
Powdered Chow Days 3–4	F_1,23_ = 0.28, *p* = 0.60	F_1,23_ = 1.09, *p* = 0.31	F_1,23_ = 3.40, *p* = 0.08
Cafeteria Days 1–8	F_1,23_ < 0.01, *p* = 0.99	**F_7,161_ = 10.79, *p* < 0.01**	F_7,161_ = 0.89, *p* = 0.52
**POSTSURGERY**	**GROUP**	**DAY**	**GROUP × DAY**
Powdered Chow Days 7–8	F_1,23_ < 0.01, *p* = 0.98	F_1,23_=2.317, *p* = 0.14	F_1,23_ = 0.66, *p* = 0.43
Cafeteria Days 9–16	**F_1,23_ = 82.30, *p* < 0.01**	F_7,161_=0.50, *p* = 0.83	F_7,161_ = 0.78, *p* = 0.61
Powdered Chow Days 9–12	**F_1,23_ = 7.59, *p* = 0.01**	**F_3,69_ = 5.07, *p* < 0.01**	F_3,69_ = 2.07, *p* = 0.11

Bolded values indicate statistical significance (*p* ≤ 0.05).

**Table 3:** Total Energy Intake within Groups across Phases and Diet Conditions.

**PRESURGERY vs. POSTSURGERY**		**CONTROL**	**RYGB**
Powdered Chow Day 4 (Pre)	Powdered Chow Day 7 (Post)	t_13_ = 1.19, *p* = 0.25	t_10_ = 0.19, *p* = 0.86
Cafeteria Day 8 (Pre)	Cafeteria Day 9 (Post)	t_13_=1.18, *p* = 0.26	**t_10_ = 4.31, *p* < 0.01**
**DIET TRANSITIONS WITHIN A PHASE**		**CONTROL**	**RYGB**
Powdered Chow Day 4 (Pre)	Cafeteria Day 1 (Pre)	t_13_ = 0.49, *p* = 0.66	t_10_ = 1.14, *p* = 0.28
Powdered Chow Day 8 (Post)	Cafeteria Day 9 (Post)	**t_13_ = 7.64, *p* < 0.01**	t_10_ = 1.07, *p* = 0.31
Cafeteria Day 16 (Post)	Powdered Chow Day 9 (Post)	**t_13_ = 11.96, *p* < 0.01**	**t_10_ = 3.42, *p* < 0.01**

Bolded values indicate statistical significance (*p* ≤ 0.05).

**Table 4:** Two-way ANOVAs Comparing 22-H Meal Sizes between Groups for Each Diet Condition.

**PRESURGERY**	**GROUP**	**DAY**	**GROUP × DAY**
Powdered Chow Days 3–4	F_1,23_ < 0.01, *p* = 0.95	F_1,23_ = 2.06, *p* = 0.17	F_1,23_ = 0.47, *p* = 0.50
Cafeteria Days 1–8	F_1,23_ < 0.01, *p* = 0.94	**F_7,161_ = 4.68, *p* < 0.01**	F_7,161_ = 0.77, *p* = 0.62
**POSTSURGERY**	**GROUP**	**DAY**	**GROUP × DAY**
Powdered Chow Days 7–8	F_1,23_ = 0.06, *p* = 0.81	F_1,23_ = 1.31, *p* = 0.26	F_1,23_ = 0.79, *p* = 0.38
Cafeteria Days 9–16	**F_1,23_ = 67.09, *p* < 0.01**	**F_7,161_ = 2.29, *p* = 0.03**	**F_7,161_ = 2.14, *p* = 0.04**
Powdered Chow Days 9–12	F_1,23_ = 0.49, *p* = 0.49	**F_3,69_ = 5.96, *p* < 0.01**	F_3,69_ = 2.07, *p* = 0.11

Bolded values indicate statistical significance (*p* < 0.05).

**Table 5:** Paired *t*-tests Comparing 22-H Meal Sizes within Groups across Diets.

**PRESURGERY vs. POSTSURGERY**		**CONTROL**	**RYGB**
Powdered Chow Day 4 (Pre)	Powdered Chow Day 7 (Post)	t_13_ = 1.19, *p* = 0.25	t_10_ = 0.19, *p* = 0.86
Cafeteria Day 8 (Pre)	Cafeteria Day 9 (Post)	t_13_ = 2.53, *p* = 0.06	t_10_ = 1.48, *p* = 0.17
**DIET TRANSITIONS WITHIN A PHASE**		**CONTROL**	**CONTROL**
Powdered Chow Day 4 (Pre)	Cafeteria Day 1 (Pre)	t_13_ = 0.21, *p* = 0.84	t_10_ = 0.06, *p* = 0.95
Powdered Chow Day 8 (Post)	Cafeteria Day 9 (Post)	**t_13_ = 5.33, *p* < 0.01**	**t_10_ = 2.94, *p* = 0.01**
Cafeteria Day 16 (Post)	Powdered Chow Day 9 (Post)	**t_13_ = 10.90, *p* < 0.01**	**t_10_ = 5.01, *p* < 0.01**

Bolded values indicate statistical significance (*p* ≤ 0.05).

**Table 6:** Two-way ANOVAs Comparing 22-H Meal Number between Groups for Each Diet Condition.

**PRESURGERY**	**GROUP**	**DAY**	**GROUP × DAY**
Powdered Chow Days 3–4	F_1,23_ = 0.90, *p* = 0.35	F_1,23_ = 1.54, *p* = 0.23	F_1,23_ = 0.21, *p* = 0.65
Cafeteria Days 1–8	F_1,23_ = 0.01, *p* = 0.91	**F_7,161_ = 3.68, *p* < 0.01**	F_7,161_ = 0.84, *p* = 0.56
**POSTSURGERY**	**GROUP**	**DAY**	**GROUP × DAY**
Powdered Chow Days 7–8	F_1,23_ = 0.06, *p* = 0.81	F_1,23_ = 0.30, *p* = 0.59	F_1,23_ = 0.19, *p* = 0.67
Cafeteria Days 9–16	F_1,23_ = 0.42, *p* = 0.52	**F_7,161_ = 3.07, *p* < 0.01**	**F_7,161_ = 3.30, *p* < 0.01**
Powdered Chow Days 9–12	**F_1,23_ = 8.81, *p* < 0.01**	F_3,69_ = 0.08, *p* = 0.97	F_3,69_ = 0.28, *p* = 0.84

Bolded values indicate statistical significance (*p* ≤ 0.05).

**Table 7:** Paired *t*-tests Comparing 22-H Meal Number within Groups across Diets.

**PRESURGERY vs. POSTSURGERY**		**CONTROL**	**RYGB**
Powdered Chow Day 4 (Pre)	Powdered Chow Day 7 (Post)	t_13_ = 0.14, *p* = 0.89	t_10_ = 0.83, *p* = 0.43
Cafeteria Day 8 (Pre)	Cafeteria Day 9 (Post)	t_13_ = 0.76, *p* = 0.46	**t_10_ = 5.52, *p* < 0.01**
**DIET TRANSITIONS WITHIN A PHASE**		**CONTROL**	**CONTROL**
Powdered Chow Day 4 (Pre)	Cafeteria Day 1 (Pre)	t_13_ = 0.43, *p* = 0.68	t_10_ = 0.91, *p* = 0.39
Powdered Chow Day 8 (Post)	Cafeteria Day 9 (Post)	t_13_ = 1.57, *p* = 0.14	**t_10_ = 2.74, *p* = 0.02**
Cafeteria Day 16 (Post)	Powdered Chow Day 9 (Post)	**t_13_ = 3.20, *p* < 0.01**	t_10_ = 0.55, *p* = 0.60

Bolded values indicate statistical significance (*p* ≤ 0.05).

**Table 8:** Two-way ANOVAs Comparing 22-H Meal Duration between Groups for Each Diet Condition.

**PRESURGERY**	**GROUP**	**DAY**	**GROUP × DAY**
Powdered Chow Days 3–4	F_1,23_ = 0.17, *p* = 0.68	F_1,23_ = 0.39, *p* = 0.53	F_1,23_ = 0.99, *p* = 0.33
Cafeteria Days 1–8	F_1,23_ < 0.01, *p* = 0.98	F_7,161_ = 1.13, *p* = 0.35	F_7,161_ = 0.93, *p* = 0.48
**POSTSURGERY**	**GROUP**	**DAY**	**GROUP × DAY**
Powdered Chow Days 7–8	F_1,23_ < 0.01, *p* = 0.98	F_1,23_ = 0.16, *p* = 0.69	F_1,23_ < 0.01, *p* = 0.96
Cafeteria Days 9–16	F_1,23_ = 1.23, *p* = 0.28	F_7,161_ = 1.20, *p* = 0.31	F_7,161_ = 0.84, *p* = 0.56
Powdered Chow Days 9–12	F_1,23_ = 2.02, *p* = 0.17	F_3,69_ = 0.50, *p* = 0.68	F_3,69_ = 0.46, *p* = 0.71

Bolded values indicate statistical significance (*p* ≤ 0.05).

**Table 9:** Paired *t*-tests Comparing 22-H Meal Duration within Groups across Diets.

**PRESURGERY vs. POSTSURGERY**		**CONTROL**	**RYGB**
Powdered Chow Day 4 (Pre)	Powdered Chow Day 7 (Post)	t_13_ = 1.33, *p* = 0.15	t_10_ = 1.10, *p* = 0.30
Cafeteria Day 8 (Pre)	Cafeteria Day 9 (Post)	t_13_ = 1.44, *p* = 0.17	**t_10_ = 2.39, *p* = 0.04**
**DIET TRANSITIONS WITHIN A PHASE**		**CONTROL**	**CONTROL**
Powdered Chow Day 4 (Pre)	Cafeteria Day 1 (Pre)	t_13_ = 2.01, *p* = 0.06	t_10_ = 1.94, *p* = 0.08
Powdered Chow Day 8 (Post)	Cafeteria Day 9 (Post)	t_13_ = 1.58, *p* = 0.14	**t_10_ = 3.05, *p* = 0.01**
Cafeteria Day 16 (Post)	Powdered Chow Day 9 (Post)	**t_13_ = 2.32, *p* = 0.04**	t_10_ = 2.00, *p* = 0.09

Bolded values indicate statistical significance (*p* ≤ 0.05).

**Table 10:** Two-way ANOVAs Comparing 22-H Meal Eating Rate between Groups for Each Diet Condition.

**PRESURGERY**	**GROUP**	**DAY**	**GROUP × DAY**
Powdered Chow Days 3–4	F_1,23_ = 0.18, *p* = 0.68	F_1,23_ = 1.11, *p* = 0.30	F_1,23_ = 0.27, *p* = 0.61
Cafeteria Days 1–8	F_1,23_ = 0.02, *p* = 0.91	**F_7,161_ = 3.16, *p* < 0.01**	F_7,161_ = 1.06, *p* = 0.39
**POSTSURGERY**	**GROUP**	**DAY**	**GROUP × DAY**
Powdered Chow Days 7–8	F_1,23_ = 0.10, *p* = 0.76	F_1,23_ = 2.00, *p* = 0.17	F_1,23_ = 0.16, *p* = 0.69
Cafeteria Days 9–16	**F_1,23_ = 32.27, *p* < 0.01**	**F_7,161_ = 3.34, *p* < 0.01**	**F_7,161_ = 3.41, *p* < 0.01**
Powdered Chow Days 9–12	F_1,23_ = 0.04, *p* = 0.84	F_3,69_ = 1.02, *p* = 0.39	F_3,69_ = 1.10, *p* = 0.36

Bolded values indicate statistical significance (*p* ≤ 0.05).

**Table 11:** Paired *t*-tests Comparing 22-H Meal Eating Rate within Groups across Diets.

**PRESURGERY vs. POSTSURGERY**		**CONTROL**	**RYGB**
Powdered Chow Day 4 (Pre)	Powdered Chow Day 7 (Post)	t_13_ = 0.96, *p* = 0.36	t_10_ = 1.41, *p* = 0.19
Cafeteria Day 8 (Pre)	Cafeteria Day 9 (Post)	**t_13_ = 3.39, *p* < 0.01**	t_10_ = 1.94, *p* = 0.08
**DIET TRANSITIONS WITHIN A PHASE**		**CONTROL**	**CONTROL**
Powdered Chow Day 4 (Pre)	Cafeteria Day 1 (Pre)	**t_13_ = 2.77, *p* = 0.02**	**t_10_ = 3.17, *p* < 0.05**
Powdered Chow Day 8 (Post)	Cafeteria Day 9 (Post)	**t_13_ = 10.88, *p* < 0.01**	**t_10_ = 4.90, *p* < 0.01**
Cafeteria Day 16 (Post)	Powdered Chow Day 9 (Post)	**t_13_ = 9.10, *p* < 0.01**	**t_10_ = 3.30, *p* < 0.01**

Bolded values indicate statistical significance (*p* ≤ 0.05).

**Table 12:** Two-way ANOVAs Comparing 22-H Intermeal Interval between Groups for Each Diet Condition.

**PRESURGERY**	**GROUP**	**DAY**	**GROUP × DAY**
Powdered Chow Days 3–4	F_1,23_ = 0.58, *p* = 0.46	**F_1,23_ = 10.19, *p* < 0.01**	F_1,23_ = 0.27, *p* = 0.61
Cafeteria Days 1–8	F_1,23_ = 0.72, *p* = 0.41	F_7,161_ = 1.65, *p* = 0.13	F_7,161_ = 0.88, *p* = 0.52
**POSTSURGERY**	**GROUP**	**DAY**	**GROUP × DAY**
Powdered Chow Days 7–8	F_1,23_ = 1.92, *p* = 0.18	F_1,23_ = 0.11, *p* = 0.74	F_1,23_ = 0.53, *p* = 0.47
Cafeteria Days 9–16	F_1,23_ = 0.13, *p* = 0.72	F_7,161_ = 0.83, *p* = 0.57	**F_7,161_ = 2.09, *p* < 0.05**
Powdered Chow Days 9–12	F_1,23_ = 0.34, *p* = 0.57	F_3,69_ = 0.25, *p* = 0.86	F_3,69_ = 0.80, *p* = 0.50

Bolded values indicate statistical significance (*p* ≤ 0.05).

**Table 13:** Paired *t*-tests Comparing 22-H Intermeal Interval within Groups across Diets.

**PRESURGERY vs. POSTSURGERY**		**CONTROL**	**RYGB**
Powdered Chow Day 4 (Pre)	Powdered Chow Day 7 (Post)	t_13_ = 1.09, *p* = 0.29	t_10_ = 0.27, *p* = 0.79
Cafeteria Day 8 (Pre)	Cafeteria Day 9 (Post)	t_13_ = 0.48, *p* = 0.64	t_10_ = 1.68, *p* = 0.13
**DIET TRANSITIONS WITHIN A PHASE**		**CONTROL**	**CONTROL**
Powdered Chow Day 4 (Pre)	Cafeteria Day 1 (Pre)	**t_13_ = 3.7, *p* < 0.01**	t_10_ = 2.25, *p* = 0.05
Powdered Chow Day 8 (Post)	Cafeteria Day 9 (Post)	t_13_ = 0.50, *p* = 0.62	t_10_ = 1.57, *p* = 0.15
Cafeteria Day 16 (Post)	Powdered Chow Day 9 (Post)	t_13_ = 1.27, *p* = 0.22	t_10_ = 0.09, *p* = 0.93

Bolded values indicate statistical significance (*p* ≤ 0.05).

**Table 14:** Two-way ANOVAs Comparing Meal Sizes for Lights-On and Lights-Off Meals between Groups for Each Diet Condition.

**PRESURGERY**	**LIGHTS-ON**			**LIGHTS-OFF**		
	**GROUP**	**DAY**	**G × D**	**GROUP**	**DAY**	**G × D**
Powdered Chow Days 3–4	F_1,23_ = 0.07, *p* = 0.80	F_1,23_ = 3.88, *p* = 0.06	F_1,23_ = 0.46, *p* = 0.51	F_1,23_ = 0.03, *p* = 0.87	F_1,23_ = 2.13, *p* = 0.16	F_1,23_ = 0.66, *p* = 0.43
Cafeteria Days 1–8	F_1,23_ = 0.02, *p* = 0.90	**F_7,161_ = 4.20, *p* < 0.01**	**F_7,161_ = 0.50, *p* = 0.83**	F_1,23_ = 0.43, *p* = 0.52	**F_7,161_ = 3.13, *p* < 0.01**	**F_7,161_ = 1.26, *p* = 0.27**
**POSTSURGERY**	**LIGHTS-ON**			**LIGHTS-OFF**		
	**GROUP**	**DAY**	**G × D**	**GROUP**	**DAY**	**G × D**
Powdered Chow Days 7–8	F_1,23_ = 0.45, *p* = 0.51	F_1,23_ = 0.30, *p* = 0.59	F_1,23_<0.01, *p* = 0.99	F_1,23_ = 0.02, *p* = 0.89	F_1,23_ = 3.10, *p* = 0.09	F_1,23_ = 1.48, *p* = 0.24
Cafeteria Days 9–16	**F_1,23_ = 28.20, *p* < 0.01**	**F_7,161_ = 9.88, *p* < 0.01**	**F_7,161_ = 2.71, *p* = 0.01**	**F_1,23_ = 60.54, *p* < 0.01**	**F_7,161_ = 1.35, *p* = 0.23**	**F_7,161_ = 1.25, *p* = 0.28**
Powdered Chow Days 9–12	F_1,23_ = 0.08, *p* = 0.78	F_3,69_ = 2.05, *p* = 0.12	F_3,69_ = 1.28, *p* = 0.29	F_1,23_ = 0.03, *p* = 0.86	**F_3,69_ = 3.77, *p* = 0.01**	F_3,69_ = 2.72, *p* > 0.05

Bolded values indicate statistical significance (*p* ≤ 0.05).

**Table 15:** Two-way ANOVAs Comparing Lights-On to Lights-Off Meal Sizes within Groups for Each Diet Condition.

**PRESURGERY**	**CONTROL**			**RYGB**		
	**LIGHTS**	**DAY**	**L × D**	**LIGHTS**	**DAY**	**L × D**
Powdered Chow Days 3–4	**F_1,13_ = 32.51, *p* < 0.01**	F_1,13_ = 2.21, *p* = 0.17	F_1,13_ = 0.10, *p* = 0.76	**F_1,10_ = 14.32, *p* < 0.01**	F_1,10_ = 1.55, *p* = 0.25	F_1,10_ = 0.97, *p* = 0.36
Cafeteria Days 1–8	F_1,13_ = 1.213, *p* = 0.29	**F_7,98_ = 2.19, *p* = 0.04**	F_7,98_ = 1.70, *p* = 0.12	F_1,10_ = 0.02, *p* = 0.90	**F_7,63_ = 3.73, *p* < 0.01**	F_7,63_ = 1.56, *p* = 0.16
**POSTSURGERY**	**CONTROL**			**RYGB**		
	**LIGHTS**	**DAY**	**L × D**	**LIGHTS**	**DAY**	**L × D**
Powdered Chow Days 7–8	**F_1,13_ = 4.45, *p* < 0.05**	F_1,13_ = 0.05, *p* = 0.83	F_1,13_ = 0.43, *p* = 0.53	**F_1,10_ = 4.63, *p* = 0.04**	F_1,10_ = 1.85, *p* = 0.21	F_1,10_ = 2.01, *p* = 0.19
Cafeteria Days 9–16	F_1,13_ = 1.54, *p* = 0.24	F_7,98_ = 1.17, *p* = 0.33	**F_7,98_ = 5.33, *p* < 0.01**	**F_1,10_ = 5.79, *p* = 0.04**	**F_7,63_ = 12.95, *p* < 0.01**	**F_7,63_ = 11.75, *p* < 0.01**
Powdered Chow Days 9–12	**F_1,23_ = 6.44, *p* = 0.03**	F_3,42_ = 0.37, *p* = 0.78	F_3,42_ = 0.44, *p* = 0.73	F_1,23_ = 3.91, *p* = 0.08	**F_3,27_ = 6.09, *p* < 0.01**	F_3,27_ = 0.57, *p* = 0.64

Bolded values indicate statistical significance (*p* ≤ 0.05).

**Table 16:** Two-way ANOVAs Comparing Meal Number for Lights-On and Lights-Off Meals between Groups for Each Diet Condition.

**PRESURGERY**	**LIGHTS-ON**			**LIGHTS-OFF**		
	**GROUP**	**DAY**	**G × D**	**GROUP**	**DAY**	**G × D**
Powdered Chow Days 3–4	F_1,23_ = 1.28, *p* = 0.27	F_1,23_ = 0.08, *p* = 0.79	F_1,23_ = 0.68, *p* = 0.42	F_1,23_ = 1.33, *p* = 0.29	F_1,23_ = 3.19, *p* = 0.09	F_1,23_ = 0.03, *p* = 0.87
Cafeteria Days 1–8	F_1,23_ = 0.36, *p* = 0.55	**F_7,161_ = 5.54, *p* < 0.01**	F_7,161_ = 0.96, *p* = 0.50	F_1,23_ = 0.04, *p* = 0.84	F_7,161_ = 2.04, *p* > 0.05	F_7,161_ = 1.08, *p* = 0.38
**POSTSURGERY**	**LIGHTS-ON**			**LIGHTS-OFF**		
	**GROUP**	**DAY**	**G × D**	**GROUP**	**DAY**	**G × D**
Powdered Chow Days 7–8	F_1,23_ = 1.15, *p* = 0.30	F_1,23_ = 0.84, *p* = 0.37	F_1,23_ = 0.05, *p* = 0.82	F_1,23_ = 0.09, *p* = 0.77	F_1,23_ = 0.02, *p* = 0.87	F_1,23_ = 0.52, *p* = 0.48
Cafeteria Days 9–16	**F_1,23_ = 13.25, *p* < 0.01**	**F_7,161_ = 3.10, *p* < 0.01**	**F_7,161_ = 2.71, *p* = 0.01**	**F_1,23_ = 5.18, *p* = 0.03**	F_7,161_ = 1.35, *p* = 0.30	F_7,161_ = 2.01, *p* > 0.05
Powdered Chow Days 9–12	**F_1,23_ = 10.76, *p* < 0.01**	F_3,69_ = 0.40, *p* = 0.76	F_3,69_ = 0.40, *p* = 0.76	**F_1,23_ = 6.19, *p* = 0.02**	F_3,69_ = 0.13, *p* = 0.94	F_3,69_ = 0.24, *p* = 0.87

Bolded values indicate statistical significance (*p* ≤ 0.05).

**Table 17:** Two-way ANOVAs Comparing Lights-On to Lights-Off Meal Number within Groups for Each Diet Condition.

**PRESURGERY**	**CONTROL**			**RYGB**		
	**LIGHTS**	**DAY**	**L × D**	**LIGHTS**	**DAY**	**L × D**
Powdered Chow Days 3–4	**F_1,13_ *=* 149.42, *p* < 0.01**	F_1,13_ *=* 2.33, *p =* 0.15	F_1,13_ *=* 0.46, *p =* 0.51	**F_1,10_ *=* 211.60, *p* < 0.01**	F_1,10_ *=* 0.19, *p =* 0.67	F_1,10_ *=* 1.48, *p =* 0.26
Cafeteria Days 1–8	**F_1,13_ *=* 137.38, *p* < 0.01**	**F_7,98_ *=* 2.86, *p* < 0.01**	**F_7,98_ *=* 2.89, *p* < 0.01**	**F_1,10_ *=* 182.12, *p* < 0.01**	F_7,63_ *=* 1.96, *p =* 0.07	F_7,63_ *=* 1.17, *p =* 0.33
**POSTSURGERY**	**CONTROL**			**RYGB**		
	**LIGHTS**	**DAY**	**L × D**	**LIGHTS**	**DAY**	**L × D**
Powdered Chow Days 7–8	**F_1,13_ *=* 258.26, *p* < 0.01**	F_1,13_ *=* 0.15, *p =* 0.71	F_1,13_ *=* 0.19, *p =* 0.67	**F_1,10_ *=* 202.50, *p* < 0.01**	F_1,10_ *=* 0.37, *p =* 0.56	F_1,10_ *=* 0.38, *p =* 0.56
Cafeteria Day 9–16	**F_1,13_ *=* 411.04, *p* < 0.01**	F_7,98_ *=* 0.24, *p =* 0.97	F_7,98_ *=* 0.84, *p =* 0.56	**F_1,10_ *=* 129.36, *p* < 0.01**	**F_7,63_ *=* 4.531, *p* < 0.01**	**F_7,63_ *=* 2.23, *p =* 0.04**
Powdered Chow Days 9–12	**F_1,23_ *=* 167.47, *p* < 0.01**	F_3,42_ *=* 0.18, *p =* 0.91	F_3,42_ *=* 0.40, *p =* 0.75	**F_1,23_ *=* 147.65, *p* < 0.01**	F_3,27_ *=* 0.25, *p =* 0.86	F_3,27_ *=* 0.13, *p =* 0.94

Bolded values indicate statistical significance (*p* ≤ 0.05).

**Table 18:** Two-way ANOVAs Comparing Meal Eating Rate for Lights-On and Lights-Off Meals between Groups for Each Diet Condition.

**PRESURGERY**	**LIGHTS-ON**			**LIGHTS-OFF**		
	**GROUP**	**DAY**	**G × D**	**GROUP**	**DAY**	**G × D**
Powdered Chow Days 3–4	F_1,23_ *=* 0.02, *p =* 0.90	F_1,23_ *=* 0.38, *p =* 0.55	F_1,23_ *=* 1.39, *p =* 0.25	F_1,23_ *=* 0.07, *p =* 0.79	F_1,23_ *=* 2.82, *p =* 0.11	F_1,23_ *=* 0.73, *p =* 0.40
Cafeteria Days 1–8	F_1,23_ *=* 0.04, *p =* 0.85	**F_7,161_ *=* 2.81, *p* < 0.01**	F_7,161_ *=* 1.08, *p =* 0.38	F_1,23_ *=* 0.15, *p =* 0.70	F_7,161_ *=* 1.61, *p =* 0.14	F_7,161_ *=* 0.76, *p =* 0.62
**POSTSURGERY**	**LIGHTS-ON**			**LIGHTS-OFF**		
	**GROUP**	**DAY**	**G × D**	**GROUP**	**DAY**	**G × D**
Powdered Chow Days 7–8	F_1,23_ *=* 0.05, *p =* 0.82	F_1,23_ *=* 0.56, *p =* 0.46	**F_1,23_ *=* 5.54, *p =* 0.03**	F_1,23_ *=* 0.08, *p =* 0.78	F_1,23_ *=* 3.03, *p =* 0.10	F_1,23_ *=* 1.11, *p =* 0.30
Cafeteria Day 9–16	**F_1,23_ *=* 34.06, *p* < 0.01**	F_7,161_ *=* 0.97, *p =* 0.46	F_7,161_ *=* 1.89, *p =* 0.08	**F_1,23_ *=* 45.91, *p* < 0.01**	F_7,161_ *=* 0.90, *p =* 0.51	F_7,161_ *=* 0.46, *p =* 0.44
Powdered Chow Days 9–12	F_1,23_ *=* 1.95, *p =* 0.18	F_3,69_ *=* 1.38, *p =* 0.26	F_3,69_ *=* 0.77, *p =* 0.51	F_1,23_ *=* 0.59, *p =* 0.45	F_3,69_ *=* 2.50, *p =* 0.07	F_3,69_ *=* 1.27, *p =* 0.29

Bolded values indicate statistical significance (*p* ≤ 0.05).

**Table 19:** Two-way ANOVAs Comparing Lights-On to Lights-Off Meal Eating Rate within Groups for Each Diet Condition.

**PRESURGERY**	**CONTROL**			**RYGB**		
	**LIGHTS**	**DAY**	**L × D**	**LIGHTS**	**DAY**	**L × D**
Powdered Chow Days 3–4	F_1,13_ = 0.11, *p* = 0.74	F_1,13_ = 0.01, *p* = 0.92	F_1,13_ = 1.26, *p* = 0.29	F_1,10_ = 0.03, *p* = 0.87	F_1,10_ = 1.30, *p* = 0.29	F_1,10_ = 0.27, *p* = 0.62
Cafeteria Days 1–8	**F_1,13_ = 36.65, *p* < 0.01**	F_7,98_ = 2.00, *p* = 0.06	F_7,98_ = 1.32, *p* = 0.25	**F_1,10_ = 35.89, *p* < 0.01**	F_7,63_ = 1.45, *p* = 0.06	F_7,63_ = 2.03, *p* = 0.07
**POSTSURGERY**	**CONTROL**			**RYGB**		
	**LIGHTS**	**DAY**	**L × D**	**LIGHTS**	**DAY**	**L × D**
Powdered Chow Days 7–8	F_1,13_ = 0.24, *p* = 0.63	F_1,13_ = 0.32, *p* = 0.58	F_1,13_ = 0.74, *p* = 0.41	F_1,10_ = 0.01, *p* = 0.91	F_1,10_ = 0.08, *p* = 0.79	F_1,10_ = 1.73, *p* = 0.23
Cafeteria Day 9-16	**F_1,13_ = 55.31, *p* < 0.01**	F_7,98_ = 0.49, *p* = 0.84	F_7,98_ = 0.92, *p* = 0.50	F_1,10_ = 0.59, *p* = 0.46	**F_7,63_ = 9.89, *p* < 0.01**	**F_7,63_ = 3.83, *p* < 0.01**
Powdered Chow Days 9–12	F_1,23_ = 1.26, *p* = 0.29	F_3,42_ = 2.30, *p* = 0.10	F_3,42_ = 0.41, *p* = 0.75	F_1,23_ = 1.42, *p* = 0.26	F_3,27_ = 0.27, *p* = 0.85	F_3,27_ = 1.99, *p* = 0.14

Bolded values indicate statistical significance (*p* ≤ 0.05).

**Table 20:** Two-way ANOVAs Comparing Intermeal Interval for Lights-On and Lights-Off Meals between Groups for Each Diet Condition.

**PRESURGERY**	**LIGHTS-ON**			**LIGHTS-OFF**		
	**GROUP**	**DAY**	**G × D**	**GROUP**	**DAY**	**G × D**
Powdered Chow Days 3–4	F_1,23_ = 0.24, *p* = 0.63	F_1,23_ = 1.09, *p* = 0.31	F_1,23_ = 0.11, *p* = 0.75	F_1,23_ = 0.08, *p* = 0.79	F_1,23_ < 0.01, *p* = 0.93	F_1,23_ = 0.06, *p* = 0.81
Cafeteria Days 1–8	F_1,23_ = 0.16, *p* = 0.69	F_7,161_ = 1.79, *p* = 0.09	F_7,161_ = 0.23, *p* = 0.98	F_1,23_ = 0.11, *p* = 0.74	**F_7,161_ = 2.49, *p* = 0.02**	F_7,161_ = 1.30, *p* = 0.25
**POSTSURGERY**	**LIGHTS-ON**			**LIGHTS-OFF**		
	**GROUP**	**DAY**	**G × D**	**GROUP**	**DAY**	**G × D**
Powdered Chow Days 7–8	F_1,23_ = 0.12, *p* = 0.73	F_1,23_ = 0.08, *p* = 0.78	F_1,23_ = 0.91, *p* = 0.35	F_1,23_ = 0.46, *p* = 0.41	F_1,23_ = 0.79, *p* = 0.39	F_1,23_ = 0.09, *p* = 0.77
Cafeteria Day 9–16	**F_1,23_ = 5.33, *p* = 0.03**	F_7,161_ = 1.67, *p* = 0.12	F_7,161_ = 1.23, *p* = 0.29	F_1,23_ = 0.22, *p* = 0.65	F_7,161_ = 1.79, *p* = 0.09	F_7,161_ = 1.27, *p* = 0.27
Powdered Chow Days 9–12	**F_1,23_ = 6.37, *p* = 0.02**	F_3,69_ = 2.03, *p* = 0.12	F_3,69_ = 1.73, *p* = 0.17	F_1,23_ = 1.23, *p* = 0.28	F_3,69_ = 0.25, *p* = 0.86	F_3,69_ = 0.45, *p* = 0.72

Bolded values indicate statistical significance (*p* ≤ 0.05).

**Table 21:** Two-way ANOVAs Comparing Lights-On to Lights-Off Intermeal Intervals within Groups for Each Diet Condition.

**PRESURGERY**	**CONTROL**			**RYGB**		
	**LIGHTS**	**DAY**	**L × D**	**LIGHTS**	**DAY**	**L × D**
Powdered Chow Days 3–4	F_1,13_ = 1.42, *p* = 0.26	F_1,13_ = 0.38, *p* = 0.55	F_1,13_ = 0.29, *p* = 0.60	F_1,10_ = 0.10, *p* = 0.76	F_1,10_ = 0.25, *p* = 0.63	F_1,10_ = 1.18, *p* = 0.31
Cafeteria Days 1–8	F_1,13_ = 0.80, *p* = 0.39	F_7,98_ = 0.67, *p* = 0.70	F_7,98_ = 1.13, *p* = 0.35	F_1,10_ = 5.96, *p*>0.05	F_7,63_ = 1.08, *p* = 0.39	F_7,63_ = 1.57, *p* = 0.16
**POSTSURGERY**	**CONTROL**			**RYGB**		
	**LIGHTS**	**DAY**	**L × D**	**LIGHTS**	**DAY**	**L × D**
Powdered Chow Days 7–8	F_1,13_ = 0.07, *p* = 0.80	F_1,13_ = 0.56, *p* = 0.47	F_1,13_ = 0.27, *p* = 0.61	F_1,10_ = 0.42, *p* = 0.53	F_1,10_ = 0.27, *p* = 0.62	F_1,10_ = 0.62, *p* = 0.45
Cafeteria Day 9–16	**F_1,13_ = 19.91, *p* < 0.01**	F_7,98_ = 1.18, *p* = 0.32	F_7,98_ = 1.76, *p* = 0.10	F_1,10_ = 4.21, *p* = 0.07	F_7,63_ = 0.79, *p* = 0.60	**F_7,63_ = 2.40, *p* = 0.03**
Powdered Chow Days 9–12	F_1,23_ = 4.70, *p* = 0.06	F_3,42_ = 2.00, *p* = 0.13	F_3,42_ = 2.14, *p* = 0.12	**F_1,23_ = 22.05, *p* < 0.01**	F_3,27_ = 0.74, *p* = 0.69	F_3,27_ = 0.75, *p* = 0.53

Bolded values indicate statistical significance (*p* ≤ 0.05).

## Data Availability

The data that support the findings of this study are available from the corresponding author upon reasonable request.

## Supplementary Materials

The following are available online at https://www.mdpi.com/article/10.3390/nu13113856/s1, Figure S1: Proportion of Intake from Each Nutrient Type for Day and Night Meals. Mean (±SE) proportion of kilocalories from nonsugar carbohydrates, sugar, protein, or fat for CTL (orange bars) and RYGB (blue bars) animals for meals when lights were on (open bars), off (hatched bars), and all meals (solid bars) on the first postsurgical day of cafeteria diet (CAF-9; top) and the last day (CAF-16; bottom). Statistical results of two-way ANOVAs can be found in Table 21, Figure S2: Drinking Behaviors. Mean (±SE) number of licks (top) and interlick interval (bottom) for CTL (orange bars; *n* = 14) and RYGB (blue bars; *n* = 11) rats from the first powdered chow days (left-most panels), the final cafeteria diet (middle panels), and the final powdered chow days (right-most panels) in postsurgical testing. Inset: two-sample *t*-tests between groups. Bolded values indicate statistical significance (*p* ≤ 0.05), Figure S3: Intake, Meal Size and Number during Diestrus and Estrus. Mean (±SE) intake (A), meal number (B) and meal size (C) for both presurgical and postsurgical phases for CTL (combined SHAM and IRON groups, *n* = 14) and RYGB (*n* = 11) rats was calculated from diestrus and estrus days for each rat during the last four days of each phase. Inset: results of paired *t*-tests comparing diestrus and estrus within each group. Significant values (*p* ≤ 0.05) are in bold, Figure S4: Proportion of Intake from Each Cafeteria Diet Food Choice during Estrus and Diestrus. Mean (±SE) proportion of intake from each food for both presurgical and postsurgical phases for CTL (combined SHAM and IRON groups, *n* = 14) and RYGB (*n* = 11) rats was calculated from diestrus and estrus days for each rat during the last four days of each phase. There were no significant differences in food choices between estrus and diestrus within each group (Table S1), Figure S5: Proportion of Intake from Each Nutrient Type during Estrus and Diestrus. Mean (±SE) proportion of intake from each nutrient type for both presurgical and postsurgical phases for CTL (combined SHAM and IRON groups, *n* = 14) and RYGB (*n* = 11) rats was calculated from diestrus and estrus days for each rat during the last four days of each phase. There were no significant differences in food choices between estrus and diestrus within each group (Table S1), Figure S6: Body Mass and Body Fat Percentage. Left: Mean (±SE) body mass for every day of meal pattern monitoring for CTL (combined SHAM and IRON groups, *n* = 14) and RYGB (*n* = 11) rats for powdered chow days (PC), acclimation days for each food, and cafeteria diet days during presurgical (left) and postsurgical (right) phases. Statistically significant results from two-way mixed ANOVAs (Table S2) are indicated by horizontal lines Gray solid (group), dark gray solid (day), or gray dashed (group × day interaction). Right: Average body fat percentage with individual values, as calculated by EchoMRI after postsurgical testing, with two-sample *t*-test results inset, Figure S7: Plasma Ferritin and GLP-1 Levels. Mean (±SE) ferritin (left) and GLP-1 (right) overlaid with individual values for SHAM (white bars; *n* = 7), IRON (gray bars; *n* = 7) and RYGB (blue bars; *n* = 11) groups. Inset letters indicate group differences based on two-sample *t*-tests (Table S3), Table S1: Two-way ANOVAs Comparing Proportion of Intake by Nutrient Source across Meals with Lights were On or Off, and All Meals, Table S2: Paired *t*-tests Comparing Proportion of Kilocalories from Each Food and Nutrient Type When Animals Were in Estrus vs. Diestrus, Table S3: Two-way ANOVAs Comparing Body Mass within Each Meal Pattern Monitoring Phase, Table S4: Two-sample *t*-tests Comparing Plasma Ferritin and GLP-1 Levels.
