# Supplementary material for: Meal Patterns and Food Choices of Female Rats Fed a Cafeteria-Style Diet Are Altered by Gastric Bypass Surgery
Source: Nutrients. 2021 Oct 28;13(11):3856. doi: 10.3390/nu13113856 (PMC8623594; doi:10.3390/nu13113856)
Supplement: Supplementary file 1 [file nutrients-13-03856-s001.zip › nutrients-1383127-SI.pdf]

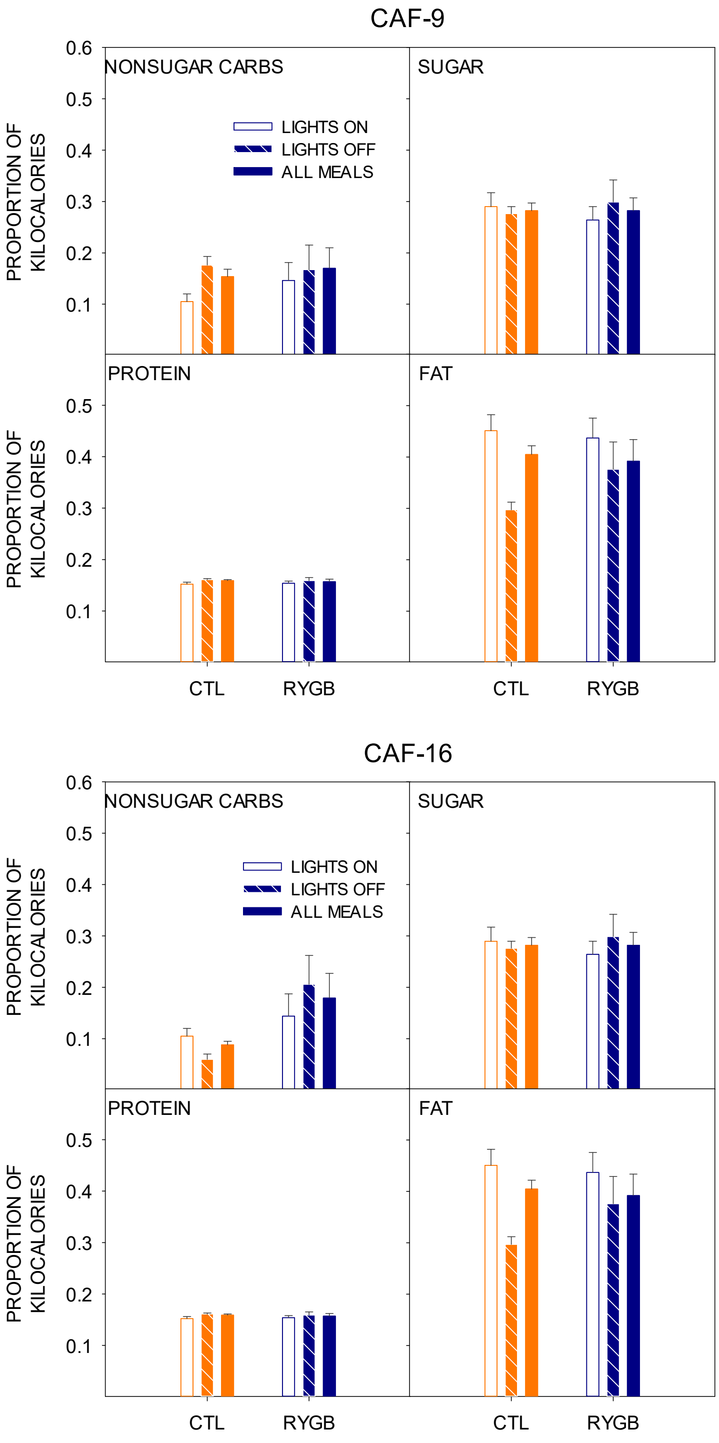

Figure S1. Proportion of Intake from Each Nutrient Type for Day and Night Meals. Mean (±SE) proportion of kilocalories from nonsugar carbohydrates, sugar, protein, or fat for CTL (orange bars) and RYGB (blue bars) animals for meals when lights were on (open bars), off (hatched bars), and all meals (solid bars) on the first postsurgical day of cafeteria diet (CAF-9; top) and the last day (CAF-16; bottom). Statistical results of two-way ANOVAs can be found in Table 22.

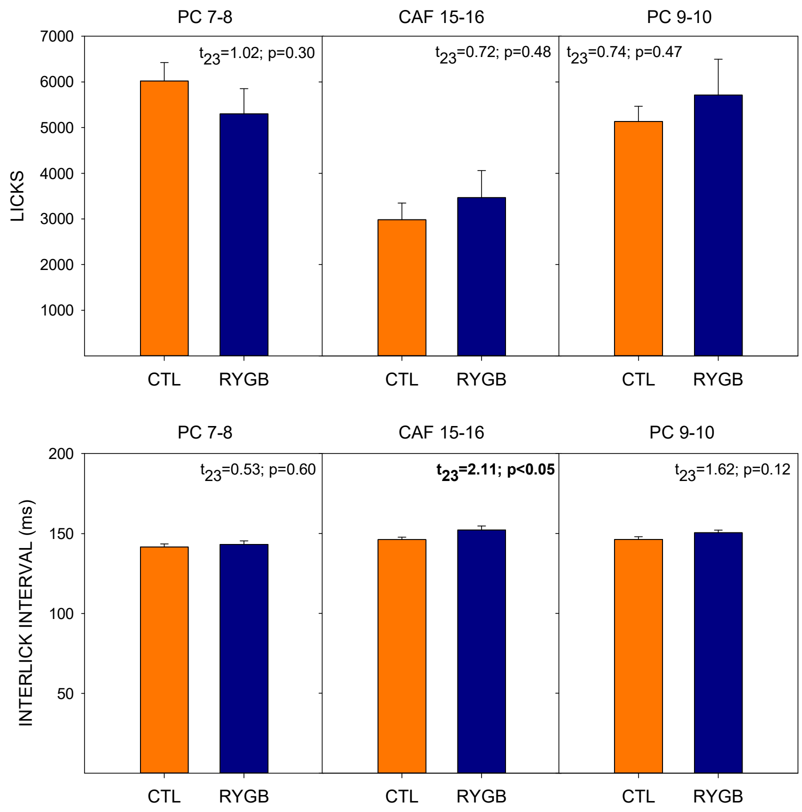

Figure S2. Drinking Behaviors. Mean (±SE) number of licks (top) and interlick interval (bottom) for CTL (orange bars; n=14) and RYGB (blue bars; n=11) rats from the first powdered chow days (left-most panels), the final cafeteria diet (middle panels), and the final powdered chow days (right-most panels) in postsurgical testing. Inset: two-sample t-tests between groups. Bolded values indicate statistical significance ($p \leq 0.05$).

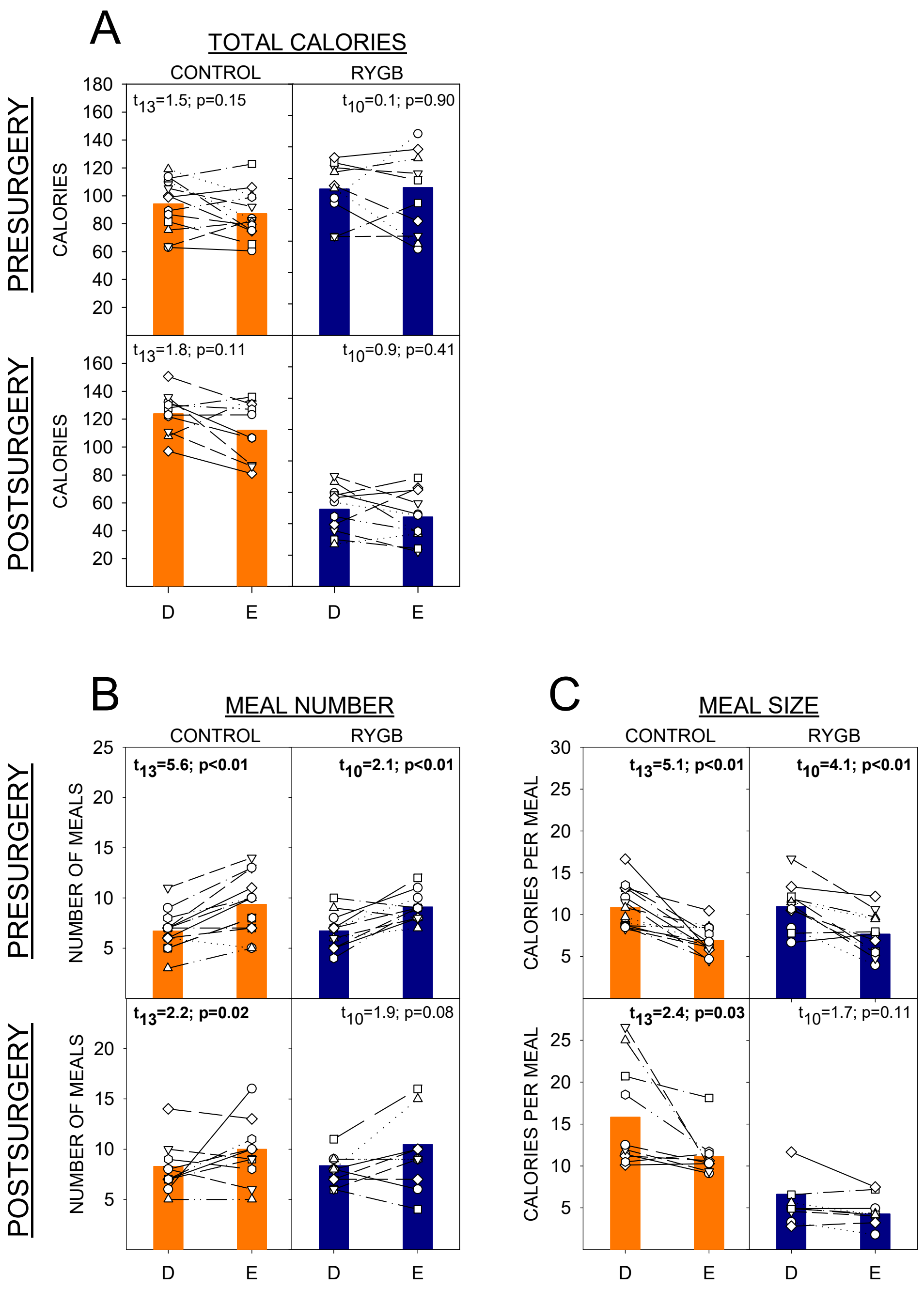

Figure S3. Intake, Meal Size and Number during Diestrus and Estrus. Mean (±SE) intake (A), meal number (B) and meal size (C) for both presurgical and postsurgical phases for CTL (combined SHAM and IRON groups, n=14) and RYGB (n=11) rats was calculated from diestrus and estrus days for each rat during the last four days of each phase. Inset: results of paired t-tests comparing diestrus and estrus within each group. Significant values ($p \leq 0.05$) are in bold.

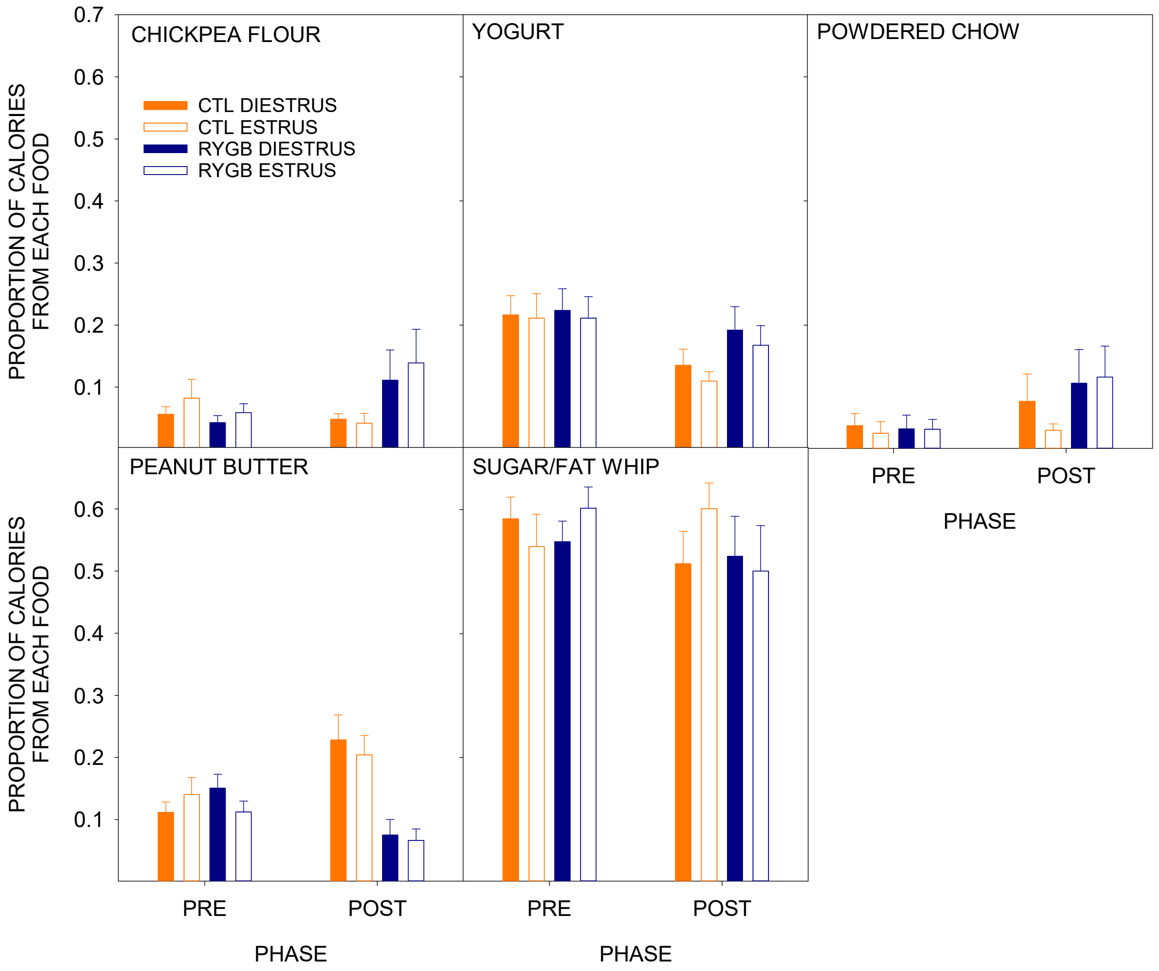

Figure S4. Proportion of Intake from Each Cafeteria Diet Food Choice during Estrus and Diestrus. Mean (±SE) proportion of intake from each food for both presurgical and postsurgical phases for CTL (combined SHAM and IRON groups, n=14) and RYGB (n=11) rats was calculated from diestrus and estrus days for each rat during the last four days of each phase. There were no significant differences in food choices between estrus and diestrus within each group (Table S1).

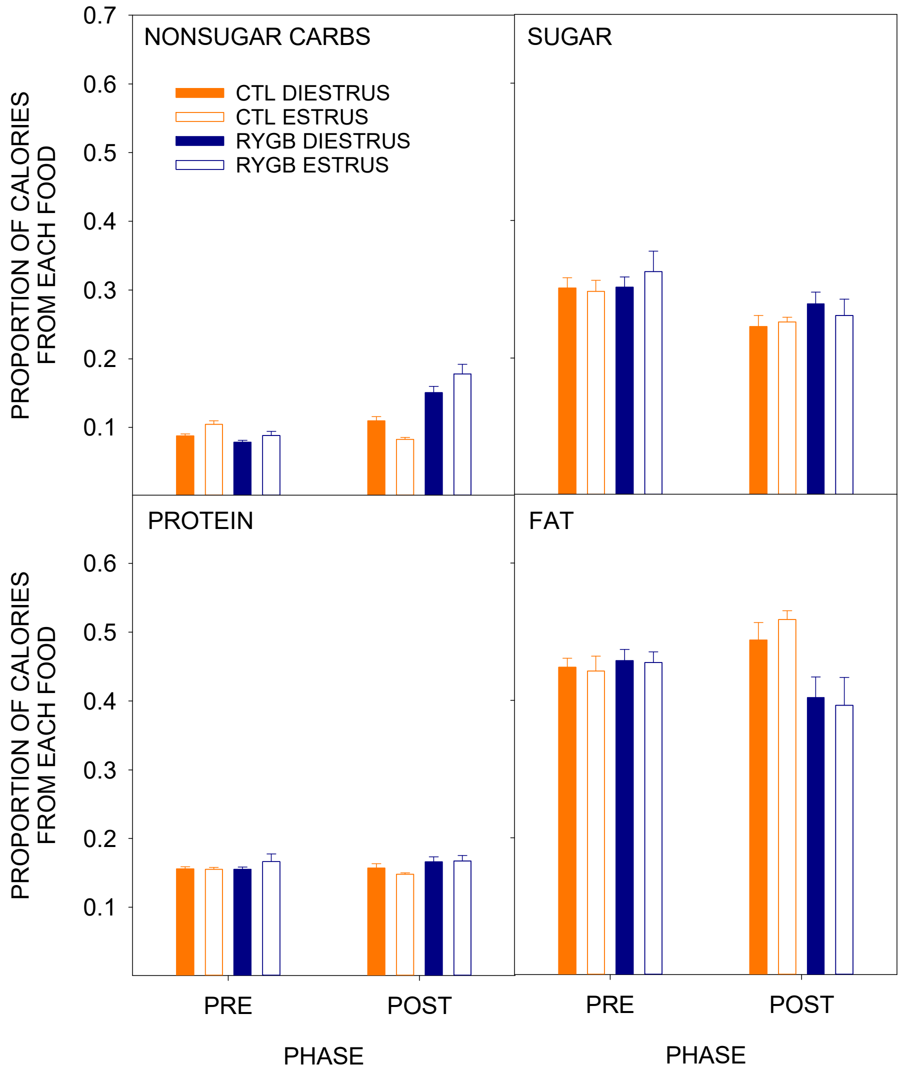

Figure S5. Proportion of Intake from Each Nutrient Type during Estrus and Diestrus. Mean (±SE) proportion of intake from each nutrient type for both presurgical and postsurgical phases for CTL (combined SHAM and IRON groups, n=14) and RYGB (n=11) rats was calculated from diestrus and estrus days for each rat during the last four days of each phase. There were no significant differences in food choices between estrus and diestrus within each group (Table S1).

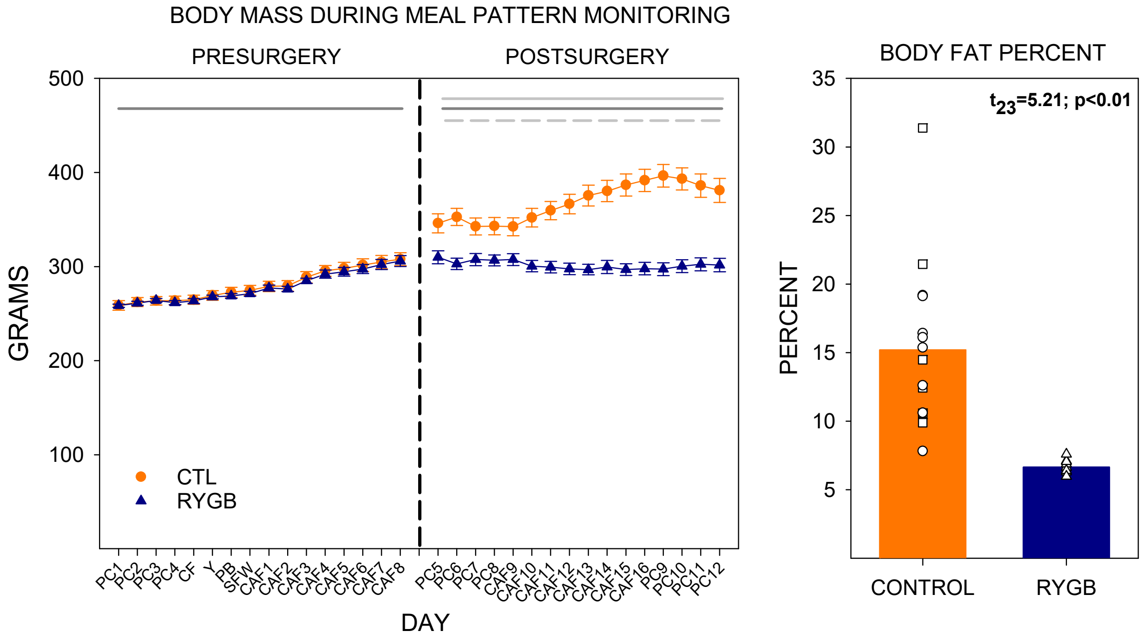

Figure S6. Body Mass and Body Fat Percentage. Left: Mean (±SE) body mass for every day of meal pattern monitoring for CTL (combined SHAM and IRON groups, n=14) and RYGB (n=11) rats for powdered chow days (PC), acclimation days for each food, and cafeteria diet days during presurgical (left) and postsurgical (right) phases. Statistically significant results from two-way mixed ANOVAs (Table S2) are indicated by horizontal lines Gray solid (group), dark gray solid (day), or gray dashed (group x day interaction). Right: Average body fat percentage with individual values, as calculated by EchoMRI after postsurgical testing, with two-sample t-test results inset.

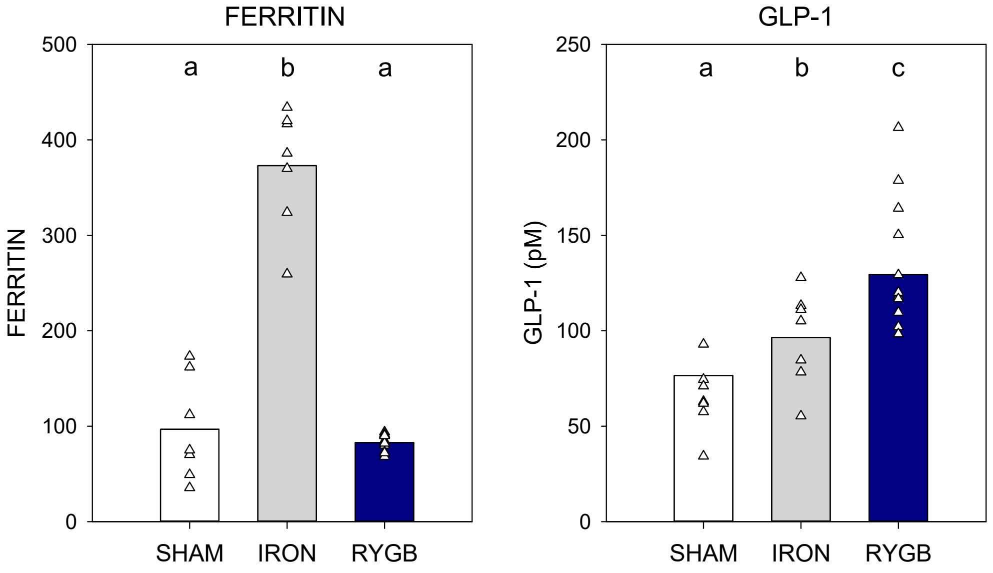

Figure S7. Plasma Ferritin and GLP-1 Levels. Mean (±SE) ferritin (left) and GLP-1 (right) overlaid with individual values for SHAM (white bars; n=7), IRON (gray bars; n=7) and RYGB (blue bars; n=11) groups. Inset letters indicate group differences based on two-sample t-tests (Table S3).

Table S1. Two-way ANOVAs Comparing Proportion of Intake by Nutrient Source across Meals with Lights were On or Off, and All Meals.

**CAFETERIA DAY 9**				
		**LIGHTS ON VS. OFF**		
	**Nonsugar Carbs**	**Sugar**	**Protein**	**Fat**
**GROUP**	$F_{1,23}$=0.18, p=0.67	$F_{1,23}$<0.01, p=0.93	$F_{1,23}$<0.01, p=0.93	$F_{1,23}$=0.09, p=0.77
**LIGHTS**	**$F_{1,23}$=4.64, p=0.04**	$F_{1,23}$=0.11, p=0.74	**$F_{1,23}$=5.00, p=0.04**	**$F_{1,23}$=5.89, p=0.02**
**GROUP x TIME**	$F_{1,23}$=1.41, p=0.25	$F_{1,23}$=0.76, p=0.39	$F_{1,23}$=0.28, p=0.60	$F_{1,23}$<0.01, p=0.95
		**LIGHTS ON VS. ALL MEALS**		
	**Nonsugar Carbs**	**Sugar**	**Protein**	**Fat**
**GROUP**	$F_{1,23}$=0.70, p=0.41	$F_{1,23}$=0.24, p=0.63	$F_{1,23}$<0.01, p=0.97	$F_{1,23}$=0.12, p=0.74
**LIGHTS**	**$F_{1,23}$=5.68, p=0.03**	$F_{1,23}$=0.05, p=0.82	**$F_{1,23}$=5.82, p=0.02**	**$F_{1,23}$=7.18, p=0.01**
**GROUP x TIME**	$F_{1,23}$=0.62, p=0.44	$F_{1,23}$=0.59, p=0.45	$F_{1,23}$=0.50, p=0.49	$F_{1,23}$<0.01, p=0.99
		**LIGHTS OFF VS. ALL MEALS**		
	**Nonsugar Carbs**	**Sugar**	**Protein**	**Fat**
**GROUP**	$F_{1,23}$<0.01, p=0.95	$F_{1,23}$=0.12, p=0.73	$F_{1,23}$=0.15, p=0.70	$F_{1,23}$=0.08, p=0.78
**LIGHTS**	$F_{1,23}$=1.77, p=0.20	$F_{1,23}$=0.23, p=0.64	$F_{1,23}$=2.83, p=0.11	$F_{1,23}$=3.02, p=0.10
**GROUP x TIME**	$F_{1,23}$=3.67, p=0.07	$F_{1,23}$=0.93, p=0.34	$F_{1,23}$=0.03, p=0.87	$F_{1,23}$=0.04, p=0.84
**CAFETERIA DAY 16**				
		**LIGHTS ON VS. OFF**		
	**Nonsugar Carbs**	**Sugar**	**PRO**	**Fat**
**GROUP**	**$F_{1,23}$=4.80, p=0.04**	$F_{1,23}$<0.01, p=0.99	**$F_{1,23}$=7.38, p=0.01**	**$F_{1,23}$=7.73, p=0.01**
**LIGHTS**	$F_{1,23}$=0.20, p=0.66	$F_{1,23}$=0.43, p=0.52	$F_{1,23}$=0.25, p=0.63	$F_{1,23}$=1.00, p=0.33
**GROUP x TIME**	**$F_{1,23}$=6.85, p=0.02**	$F_{1,23}$=0.50, p=0.49	$F_{1,23}$=0.87, p=0.36	**$F_{1,23}$=5.12, p=0.03**
		**LIGHTS ON VS. ALL MEALS**		
	**Nonsugar Carbs**	**Sugar**	**Protein**	**Fat**
**GROUP**	$F_{1,23}$=2.50, p=0.13	$F_{1,23}$<0.01, p=0.95	$F_{1,23}$=4.05, p=0.06	$F_{1,23}$=3.75, p=0.07
**LIGHTS**	$F_{1,23}$=0.71, p=0.41	$F_{1,23}$=0.42, p=0.52	$F_{1,23}$<0.01, p=0.97	$F_{1,23}$=2.55, p=0.12
**GROUP x TIME**	**$F_{1,23}$=5.65, p=0.03**	$F_{1,23}$=1.52, p=0.23	$F_{1,23}$=0.06, p=0.81	$F_{1,23}$=2.38, p=0.14
		**LIGHTS OFF VS. ALL MEALS**		
	**Nonsugar Carbs**	**Sugar**	**Protein**	**Fat**
**GROUP**	**$F_{1,23}$=6.55, p=0.02**	$F_{1,23}$=0.13, p=0.72	**$F_{1,23}$=9.32, p<0.01**	**$F_{1,23}$=9.94, p<0.01**
**LIGHTS**	$F_{1,23}$<0.01, p=0.99	$F_{1,23}$=0.35, p=0.56	$F_{1,23}$=0.99, p=0.14	$F_{1,23}$=0.13, p=0.72
**GROUP x TIME**	**$F_{1,23}$=7.05, p=0.01**	$F_{1,23}$=0.04, p=0.85	$F_{1,23}$=2.41, p=0.14	**$F_{1,23}$=6.48, p=0.02**

Bolded values indicate statistical significance (p≤0.05).

Table S2. Paired t-tests Comparing Proportion of Kilocalories from Each Food and Nutrient Type When Animals Were in Estrus vs. Diestrus.

	CONTROL		RYGB	
**FOOD PROPORTIONS**				
	**PRESURGICAL**	**POSTSURGICAL**	**PRESURGICAL**	**POSTSURGICAL**
Chickpea Flour	t_{13}=1.21, p=0.25	t_{13}=0.37, p=0.72	t_{10}=1.07, p=0.31	t_{10}=0.82, p=0.43
Yogurt	t_{13}=0.19, p=0.85	t_{13}=0.85, p=0.42	t_{10}=0.89, p=0.39	t_{10}=0.47, p=0.65
Powdered Chow	t_{13}=0.99, p=0.34	t_{13}=0.83, p=0.43	t_{10}=0.04, p=0.97	t_{10}=0.49, p=0.64
Peanut Butter	t_{13}=1.06, p=0.31	t_{13}=0.81, p=0.44	t_{10}=1.56, p=0.28	t_{10}=0.46, p=0.65
Sugar/Fat Whip	t_{13}=0.93, p=0.37	t_{13}=1.59, p=0.16	t_{10}=2.05, p=0.07	t_{10}=0.32, p=0.71
**NUTRIENT TYPES**				
	**PRESURGICAL**	**POSTSURGICAL**	**PRESURGICAL**	**POSTSURGICAL**
Carbohydrates	t_{13}=0.82, p=0.43	t_{13}=0.95, p=0.37	t_{10}=1.41, p=0.19	t_{10}=0.45, p=0.66
Sugar	t_{13}=0.48, p=0.64	t_{13}=0.35, p=0.74	t_{10}=0.85, p=0.41	t_{10}=0.83, p=0.43
Protein	t_{13}=0.19, p=0.86	t_{13}=1.10, p=0.30	t_{10}=0.99, p=0.34	t_{10}=0.34, p=0.75
Fat	t_{13}=0.34, p=0.74	t_{13}=1.04, p=0.32	t_{10}=0.24, p=0.82	t_{10}=0.45, p=0.67

Table S3. Two-way ANOVAs Comparing Body Mass within Each Meal Pattern Monitoring Phase.

	GROUP	DAY	GROUP X DAY
Presurgical Phase	$F_{1,23}$=2.30, p=0.15	**$F_{15,345}$=155.50, p<0.01**	$F_{15,345}$=0.27, p=0.98
Postsurgical Phase	**$F_{1,23}$=29.95, p<0.01**	**$F_{15,345}$=34.93, p<0.01**	**$F_{15,345}$=65.43, p<0.01**

Bolded values indicate statistical significance (p≤0.05).

Table S4. Two-sample t-tests Comparing Plasma Ferritin and GLP-1 Levels.

	RYGB	SHAM
**GLP-1**		
	RYGB	SHAM
SHAM	**t_{16}=5.67, p<0.01**	
IRON	**t_{16}=2.61, p=0.02**	**t_{12}=2.43, p=0.03**
**FERRITIN**		
	RYGB	SHAM
SHAM	t_{16}=2.31, p=0.06	
IRON	**t_{16}=17.00, p<0.01**	**t_{12}=3.46, p<0.01**

Bolded values indicate statistical significance (p≤0.05).
